# Diagnostic Performance of In‐Procedure Angiography‐Derived Quantitative Flow Reserve Compared to Pressure‐Derived Fractional Flow Reserve: The FAVOR II Europe‐Japan Study

**DOI:** 10.1161/JAHA.118.009603

**Published:** 2018-07-06

**Authors:** Jelmer Westra, Birgitte Krogsgaard Andersen, Gianluca Campo, Hitoshi Matsuo, Lukasz Koltowski, Ashkan Eftekhari, Tommy Liu, Luigi Di Serafino, Domenico Di Girolamo, Javier Escaned, Holger Nef, Christoph Naber, Marco Barbierato, Shengxian Tu, Omeed Neghabat, Morten Madsen, Matteo Tebaldi, Toru Tanigaki, Janusz Kochman, Samer Somi, Giovanni Esposito, Giuseppe Mercone, Hernan Mejia‐Renteria, Federico Ronco, Hans Erik Bøtker, William Wijns, Evald Høj Christiansen, Niels Ramsing Holm

**Affiliations:** ^1^ Department of Cardiology Aarhus University Hospital Skejby Denmark; ^2^ Cardiovascular Institute Azienda Ospedaliero‐Universitaria di Ferrara Cona Italy; ^3^ Maria Cecilia Hospital GVM Care and Research Cotignola (RA) Italy; ^4^ Department of Cardiovascular Medicine Gifu Heart Center Gifu City Japan; ^5^ Department of Cardiology Medical University of Warsaw Warszawa Poland; ^6^ Department of Cardiology Hagaziekenhuis The Hague The Netherlands; ^7^ Division of Cardiology Department of Advanced Biomedical Sciences University of Naples Federico II Naples Italy; ^8^ Azienda Ospedaliera Sant'Anna e San Sebastiano Caserta Italy; ^9^ Hospital Clinico San Carlos IDISSC Complutense University Madrid Spain; ^10^ Department of Cardiology and Angiology University of Giessen Giessen Germany; ^11^ Klinik für Kardiologie und Angiologie Essen Germany; ^12^ Emodinamica Aziendale AULSS 3 Serenissima Ospedale Dell'Angelo Mestre Italy; ^13^ Department of Clinical Epidemiology Aarhus University Hospital Skejby Denmark; ^14^ School of Biomedical Engineering Shanghai Jiao Tong University Shanghai China; ^15^ The Lambe Institute for Translational Medicine and Curam National University of Ireland Galway Galway Ireland

**Keywords:** fractional flow reserve, quantitative coronary angiography, Angiography, Diagnostic Testing, Imaging

## Abstract

**Background:**

Quantitative flow ratio (QFR) is a novel modality for physiological lesion assessment based on 3‐dimensional vessel reconstructions and contrast flow velocity estimates. We evaluated the value of online QFR during routine invasive coronary angiography for procedural feasibility, diagnostic performance, and agreement with pressure‐wire–derived fractional flow reserve (FFR) as a gold standard in an international multicenter study.

**Methods and Results:**

FAVOR II E‐J (Functional Assessment by Various Flow Reconstructions II Europe‐Japan) was a prospective, observational, investigator‐initiated study. Patients with stable angina pectoris were enrolled in 11 international centers. FFR and online QFR computation were performed in all eligible lesions. An independent core lab performed 2‐dimensional quantitative coronary angiography (2D‐QCA) analysis of all lesions assessed with QFR and FFR. The primary comparison was sensitivity and specificity of QFR compared with 2D‐QCA using FFR as a reference standard. A total of 329 patients were enrolled. Paired assessment of FFR, QFR, and 2D‐QCA was available for 317 lesions. Mean FFR, QFR, and percent diameter stenosis were 0.83±0.09, 0.82±10, and 45±10%, respectively. FFR was ≤0.80 in 104 (33%) lesions. Sensitivity and specificity by QFR was significantly higher than by 2D‐QCA (sensitivity, 86.5% (78.4–92.4) versus 44.2% (34.5–54.3); *P*<0.001; specificity, 86.9% (81.6–91.1) versus 76.5% (70.3–82.0); *P*=0.002). Area under the receiver curve was significantly higher for QFR compared with 2D‐QCA (area under the receiver curve, 0.92 [0.89–0.96] versus 0.64 [0.57–0.70]; *P*<0.001). Median time to QFR was significantly lower than median time to FFR (time to QFR, 5.0 minutes [interquartile range, –6.1] versus time to FFR, 7.0 minutes [interquartile range, 5.0–10.0]; *P*<0.001).

**Conclusions:**

Online computation of QFR in the catheterization laboratory is clinically feasible and is superior to angiographic assessment for evaluation of intermediary coronary artery stenosis using FFR as a reference standard.

**Clinical Trial Registration:**

URL: https://www.clinicaltrials.gov. Unique identifier: NCT02959814.

**Clinical Perspective**

**What Is New?**

- Quantitative flow ratio (QFR) estimates fractional flow reserve based on computation of 2 standard angiographic projections.
- Online QFR performed during invasive angiography is feasible and can be computed within the time of conventional fractional flow reserve measurement.
- QFR has superior sensitivity and specificity for detection of functional significant lesions in comparison with 2‐dimensional quantitative coronary angiography using fractional flow reserve as reference.

**What Are the Clinical Implications?**

- QFR may broaden the access to physiological lesion assessment in diagnostic catheterization laboratories and centers with low adoption of pressure‐wire–based diagnostic strategies.
- Randomized trials are required to confirm that QFR provides noninferior clinical outcome compared to assessment of intermediate coronary stenosis by pressure wire.

## Introduction

Physiological assessment is the clinical standard to guide percutaneous coronary interventions of intermediate coronary stenosis. Following the FAME (Fractional Flow Reserve Versus Angiography for Multivessel Evaluation [fractional flow reserve versus angiography for guiding percutaneous coronary intervention]) trials, the adoption of fractional flow reserve (FFR) has improved with a 16‐fold increase in FFR‐guided percutaneous coronary intervention in the United States from 2008 to 2012.^1^ Globally, the use of physiological lesion assessment remains low, with large areas performing less than 15% of eligible procedures with physiology guidance.^2^, ^3^

To further expand the use of physiological‐guided percutaneous coronary intervention, coronary computed tomography angiography– and invasive coronary angiography–based computation methods were developed for less‐invasive FFR approximation.^4^, ^5^, ^6^, ^7^, ^8^, ^9^, ^10^

Quantitative flow ratio (QFR) is a method for fast computation of FFR based on 3‐dimensional quantitative coronary angiography (3D‐QCA) and estimation of contrast flow velocity during invasive coronary angiography. The optimal approach was validated in the FAVOR (Functional Assessment by Various Flow Reconstructions) multicenter study, proving that QFR can be computed without pharmacology‐induced hyperemia.^11^ In FAVOR, QFR was computed post hoc in a core‐lab setting. The FAVOR II China study, conducted in parallel to FAVOR II Europe‐Japan (E‐J), showed a high diagnostic accuracy of in‐procedure QFR.^12^

In FAVOR II E‐J, we aimed to validate the in‐procedure feasibility and compare the diagnostic performance of QFR computation with 2‐dimensional quantitative coronary angiography (2D‐QCA) in a multicenter setting, using FFR as a reference standard.

## Methods

### Study Design

FAVOR II E‐J was a prospective, blinded, observational study with paired assessment of QFR, 2D‐QCA, and FFR performed at 11 international sites: Italy (4), The Netherlands (1), Germany (2), Poland (1), Spain (1), Japan (1), and Denmark (1). Clinicaltrials.gov identifier: NCT02959814.

### Primary Comparison

The primary comparison was sensitivity and specificity of QFR compared with 2D‐QCA to detect hemodynamically significant coronary lesions with FFR as a gold standard. For FFR and QFR, significant obstructions were defined as FFR and QFR ≤0.80 whereas >50% diameter stenosis (% DS) was used for 2D‐QCA. Sample‐size calculation and a full list of secondary comparisons are provided in Data S1 and Table S1.

### Patient Population

Patients with stable angina pectoris or patients scheduled for secondary evaluation of stenosis after acute myocardial infarction were eligible for enrollment when the angiographic inclusion criteria were met; indication for FFR measurement (at least 1 lesion with % DS 30–90 in a vessel with reference size >2.0 mm). Exclusion criteria were: acute myocardial infarction within 72 hours; severe asthma or severe chronic obstructive pulmonary disease; allergy to contrast media or adenosine; or atrial fibrillation. All inclusion and exclusion criteria are listed in Table S2.

### Ethics

The study was approved by the Central Denmark Region Committees on Biomedical Research Ethics. Approval by local or national medical ethics committees was obtained by the local or national coordinating investigators as required for the individual sites. The Danish Data Protection Agency approved the study. All enrolled patients provided written informed consent. J.W. and N.R.H. had full access to all data in the study. All authors are responsible for integrity of the analysis. The data will not be made available to other researchers for purposes of reproducing the results or replication the procedure because of competitive reasons.

### Study Procedure

#### Invasive coronary angiography

Nitroglycerine (100–200 μg IC) was administrated after acquiring the first angiographic projection. If FFR was indicated in 1 or more vessels, 2 study projections were obtained for each lesion of interest at a minimum of 12.5 frames per second. Selection of projections aimed for minimal vessel foreshortening and minimal vessel overlap by: (1) brisk, continuous and fast contrast injections and (2) no zooming and movement of the table and visualization of the entire vessel to the intended location of the pressure transducer. A table of recommended projection angles was provided for all study sites (Table S3). Images were transferred to a workstation for computation of QFR following site‐specific blinding protocols (Data S1). The remaining diagnostic invasive coronary angiography and further interventions were performed per normal clinical practice.

#### QFR computation

QFR was computed with the CE‐marked software; QAngio XA‐3D/QFR solution (Medis medical imaging system bv., Leiden, The Netherlands). An end‐diastolic frame was selected for each study projection and was used for the 3‐dimensional reconstruction of the segmented vessel. The reference vessel was constructed by fitting to healthy segments preferably proximal and distal to the lesion of interest. The following quality checks of the reference vessel reconstruction were performed: vessel tapering; good correspondence between the 2 images used for reconstruction; the reference should not follow aneurysmatic sections; and realistic proximal sizing per sex and race. The contrast frame count was performed in an angiographic run with contrast movement clearly visualized and preferably with frames from the same cardiac cycle.^13^ The detailed standard operating procedure for QFR computation is presented in Data S2. All analyses were repeated in a core‐lab setting (CardHemo, Med‐X Research Institute, Shanghai Jiao Tong University, Shanghai, China). Frame count based contrast‐QFR was used for all analysis.

#### FFR measurement

FFR was measured according to current guidelines.^14^ Volcano (San Diego, CA) or Abbott (Abbott Park, IL) pressure wires were used. Hyperemia was induced using intravenous adenosine (femoral or brachial vein infusion of adenosine at 140 μg/L/min) or intracoronary adenosine (100 μg [right coronary artery] or 200 μg [left coronary artery]). The pressure transducer location was documented angiographically for all measurements. A drift‐value within the range of 0.04 was accepted; otherwise, the procedure was repeated. For FFR values of 0.76 to 0.84, a drift value not exceeding 0.02 was required.

### Core‐Lab Waveform Analysis

FFR waveform analysis was performed at the Institute of Clinical Medicine, Aarhus University, Denmark. The observer was blinded to clinical and procedural information. Exclusion of cases with nonanalyzable FFR waveforms required massive dampening, no identification of a stable distal pressure/aortic pressure ratio during hyperemia (identical value in the same phase of the cardiac cycle over 3 beats), no drift measurement, or loss of distal pressure or aortic pressure.

### Continuous Feedback

During the enrollment period, all sites received day‐to‐day feedback from the QFR and FFR core labs on image acquisition quality, pressure wave‐form quality, and adherence to the standard operating procedure for QFR analysis.

### Statistical Analysis

Baseline characteristics and procedural characteristics were presented as count and percentages, continuous variables as mean and SD, if normally distributed, or otherwise reported as medians and interquartile range. Feasibility was calculated as the fraction of successful QFR computations of lesions with successful FFR measurements. The primary comparison was calculated as superiority for sensitivity and specificity of QFR (in‐procedure value) in comparison with 2D‐QCA (Table S1). Sensitivity and specificity for 2D‐QCA and QFR were compared using McNemar's test. Negative predictive value, positive predicate value, positive likelihood ratio, and negative likelihood ratio for 2D‐QCA and QFR were compared using generalized score statistics. Time to FFR and QFR were compared using Wilcoxon's rank test. The diagnostic performance of QFR compared with 2D‐QCA was assessed by 2‐tailed paired comparison of receiver operating characteristics curves (DeLong's method). Pearson's correlation was used to quantify the correlation between QFR and FFR. Agreement between QFR and FFR was assessed by Bland–Altman plots. Observations in patients with more than 1 study vessel were presumed independent. This assumption was evaluated by repeated analysis on a per‐patient level. If multiple measurements were performed, the lowest FFR and corresponding QFR and % DS (2D‐QCA) values were compared with per‐patient analysis. Reproducibility was assessed as interobserver variation by Bland–Altman and scatter analysis of in‐procedure QFR and core‐lab QFR. The diagnostic performance of core‐lab QFR compared with in‐procedure QFR was assessed by 2‐tailed paired comparison of receiver operating characteristics curves (DeLong's method) using FFR as a reference. Subgroup analysis for QFR accuracy was performed per FFR strata, per vessel, and for single versus tandem lesions. The diagnostic performance of 3‐dimensional quantitative coronary angiography–derived % DS and area stenosis was compared with QFR with FFR as a reference standard using receiver operating characteristics curves (DeLong's method). Analysis was performed using STATA (version 13; StataCorp LP, College Stadion, TX) and R software (R Foundation for Statistical Computing, Vienna, Austria).

## Results

**Figure 1:**
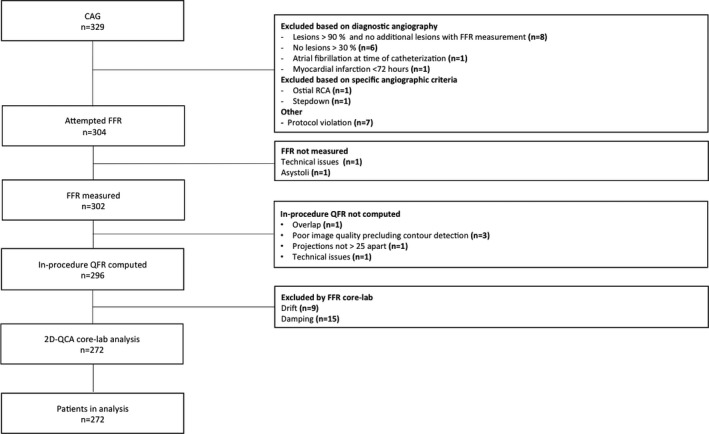
Study enrollment flow chart. FFR indicates fractional flow reserve; N, number of patients; QCA, quantitative coronary angiography; QFR, quantitative flow ratio; RCA, right coronary artery.

Three hundred twenty‐nine patients were included from February 22, 2017 to October 17, 2017 (Table S4). In‐procedure QFR was computed in 345 (96%) vessels with successful FFR measurements. After exclusion based on predefined FFR core‐lab criteria, 272 patients and 317 vessels were included in the final analysis (patient flow chart in Figure 1 and vessel‐level flow chart in Figure S1). Mean FFR was 0.83±0.09 (Figure S2), and mean % DS (2D‐QCA) was 45±10%. An FFR ≤0.80 was found in 104 (33%) vessels. Baseline and procedural characteristics are listed in Tables 1 and 2.

**Table 1:** Baseline Characteristics

Baseline Characteristics (n=272)	
Clinical	
Age, y	67±10
Male	196 (72%)
Smoking (current or past)	156 (57%)
BMI, kg/m^2^	27±5
Hypertension	201 (74%)
Hyperlipidemia	186 (68%)
Diabetes mellitus	78 (29%)
Family history of CAD	73 (27%)
Ejection fraction, %	56±10
Previous PCI	109 (40%)
Previous CABG	11 (4%)
Clinical presentation	
CCS 0	54 (20%)
CCS I	67 (25%)
CCS II	122 (45%)
CCS III	14 (5%)
CCS IV	1 (0%)
Acute myocardial infarction	6 (2%)
Other (dyspnea, arrythmia)	8 (3%)
Procedure characteristics	
Procedure time, min	43±30
Flouro time, min	10±7
Contrast use, mL	118±72

Data presented as n (%) or mean±SD. BMI indicates body mass index; CABG, coronary artery bypass surgery; CAD, coronary artery disease; CCS, Canadian Cardiovascular Society grading of angina pectoris; PCI, percutaneous coronary interventions.

**Table 2:** Lesion Characteristics

Vessel Characteristics (n=317)	
Vessel	
Left main coronary artery	4 (1%)
Left anterior descending artery	160 (50%)
Diagonal branch	5 (1%)
Left circumflex artery	50 (16%)
Obtuse marginal branch	23 (7%)
Ramus intermedius	3 (1%)
Right coronary artery	68 (22%)
Posterior descending artery	2 (1%)
Posterolateral branch	2 (1%)
Anatomy	
Diameter stenosis, %	45±10
Minimal lumen diameter, mm	1.57 (IQR, 1.27–1.90)
Reference diameter, mm	2.82 (IQR, 2.44–3.20)
Minimal lumen area, mm^2^	1.93 (IQR, 1.26–2.84)
Lesion length, mm	9.64 (IQR, 7.53–13.76)
Tandem lesions	124 (39%)
Calcified vessels	41 (13%)
Tortuous vessels	34 (11%)
Physiology	
FFR	0.83±0.09
FFR ≤0.80	104 (33%)
FFR 0.75 to 0.85	101 (32%)

Data are presented as n (%) or mean±SD. FFR indicates fractional flow reserve; IQR, interquartile range.

### Diagnostic Performance of QFR and 2D‐QCA

**Figure 2:**
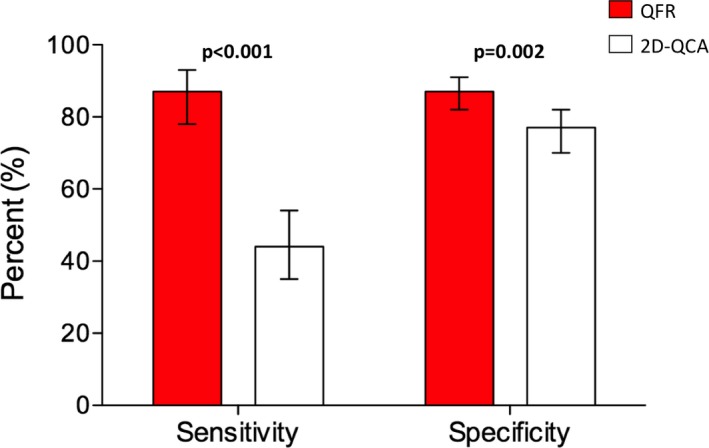
Sensitivity and specificity for QFR and 2D‐QCA with FFR as reference. QFR was superior to 2D‐QCA on sensitivity and specificity with FFR as reference standard. Diagnostic cutoffs: ≤0.80 for FFR and QFR; ≥50% DS for 2D‐QCA. 2D‐QCA indicates 2‐dimensional coronary angiography; QFR, quantitative flow ratio.

**Figure 3:**
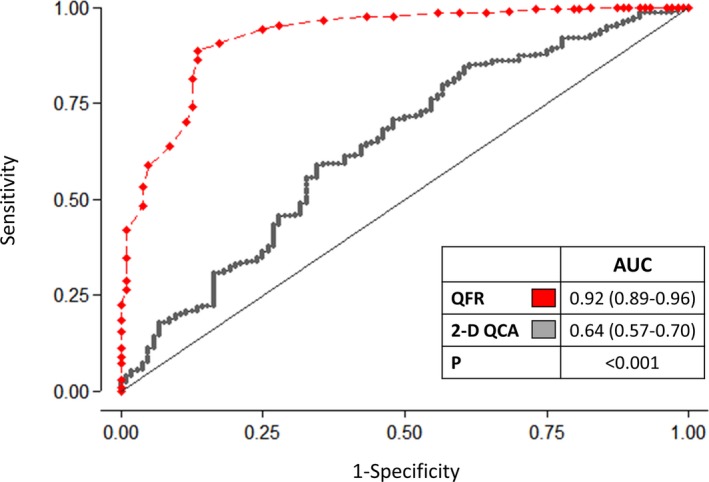
Per‐vessel level diagnostic performance. FFR≤0.80 was used as reference. 2D‐QCA indicates 2‐dimensional coronary angiography; AUC, area under the receiver operating curve; QFR, quantitative flow ratio.

Sensitivity and specificity of QFR were significantly higher than of 2D‐QCA 50% DS with FFR as a reference (sensitivity, 86.5% [95% confidence interval {CI}, 78.4–92.4] versus 44.2% [95% CI, 34.5–54.3]; *P*<0.001 and specificity, 88.9% [95% CI, 81.6–91.1] versus 76.5% [95% CI, 70.3–82.9]; *P*=0.002; Figure 2). Overall diagnostic accuracy was significantly higher for QFR compared with 2D‐QCA 50% DS using FFR ≤0.80 as a reference (86.8% versus 65.9%; *P*<0.001; Table S5). Area under receiver curve (AUC) was larger for QFR compared with 2D‐QCA with FFR as a reference (AUC, 0.92 [95% CI, 0.89–0.95] versus 0.64 [95% CI, 0.57–0.70]; *P*<0.001; Figure 3). QFR was also superior on a per‐patient level (sensitivity, 83.5% [95% CI, 74.9–90.1] versus 40.8% [95% CI, 31.2–50.9]; *P*<0.001; specificity, 83.4% [95% CI, 77.0–88.7] versus 74% [95% CI, 66.7–80.4]; *P*=0.03; and AUC, 0.91 [95% CI, 0.87–0.94] versus 0.60 [95% CI, 0.53–0.67]; *P*<0.001; Figure S3). Additional results of diagnostic comparisons are listed in Table 3.

**Table 3:** Diagnostic Performance

	QFR	2D‐QCA	*P* Value
Accuracy	86.8%	65.9%	<0.001
AUC	0.92 (0.89–0.96)	0.64 (0.57–0.70)	<0.001
Sensitivity	86.5 (78.4–92.4)	44.2 (34.5–54.3)	<0.001
Specificity	86.9 (81.6–91.1)	76.5 (70.3–82.0)	0.002
PPV	76.3 (67.6–83.6)	47.9 (37.6–58.4)	<0.001
NPV	93.0 (88.5–96.1)	73.8 (67.4–79.4)	0.001
LR (+)	6.58 (4.62–9.37)	1.88 (1.36–2.61)	<0.001
LR (−)	0.16 (0.09–0.25)	0.73 (0.61–0.88)	0.001

Comparison of QFR and 2D‐QCA with FFR as reference. Diagnostic cut‐offs: ≤0.80 for FFR and QFR; ≥50% DS for 2D‐QCA. 2D‐QCA indicates two‐dimensional quantitative coronary angiography; LR (−), negative likehood ratio; LR (+), positive likehood ratio; NPV, negative predictive value; PPV, positive predictive value; QFR, quantitative flow ratio.

### Correlation and Agreement

**Figure 4:**
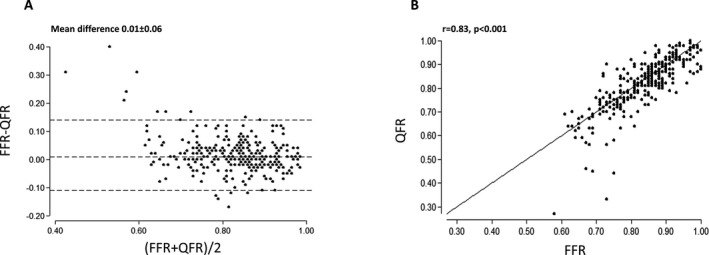
Agreement between QFR and FFR. A good correlation (A) and agreement (B) of QFR and FFR was observed. Dashed lines in Bland–Altman plot illustrate mean difference ±2 SD. FFR indicates fractional flow reserve; QFR, quantitative flow ratio.

QFR showed per‐vessel correlation (*r*=0.83; *P*<0.001) and agreement (mean difference, 0.01±0.06) with FFR (Figure 4). QFR showed per‐patient correlation (*r*=0.80; *P*<0.001) and agreement (mean difference, 0.01±0.07; Figure S4).

### Time to FFR and QFR

**Figure 5:**
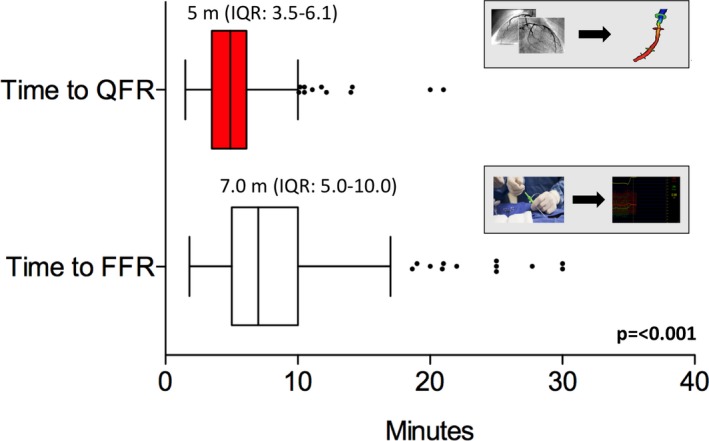
Comparison of time to FFR and time to QFR. FFR indicates fractional flow reserve; IQR, inter quartile range; m, minutes; QFR, quantitative flow ratio.

Paired assessment of time to QFR and FFR was available for 295 lesions (93%). Time to completed QFR was significantly shorter than time to completed FFR (median time to QFR, 5.0 minutes [interquartile range, 3.5–6.1] versus median time to FFR 7.0 minutes [interquartile range, 5.0–10.0]; *P*<0.001; Figure 5).

### Reproducibility

Core‐lab analysis showed per‐vessel correlation (*r*=0.73; *P*<0.001) and agreement (mean difference, −0.01±0.06) for QFR and FFR (Figure S5). We found no statistically significant difference in per‐patient AUC for in‐procedure QFR and core‐lab QFR measurements (Figure S6). Comparison of in‐procedure QFR and core‐lab QFR revealed correlation (rho, 0.83; *P*<0.001) and agreement (mean difference, −0.03±0.07; Figure S7).

### Hybrid Model Limits

QFR limits to yield specificity and sensitivity >95% with FFR as a reference were 0.77 (QFR‐treat) and 0.87 (QFR‐defer). Applying the 95% limits to this sample, use of pressure wires and adenosine could theoretically have been avoided in 64% of all measurements yielding 95% accuracy with FFR as a reference standard (Figure S8). Applying a 100% limit (QFR‐treat 0.64 and QFR‐defer 0.93) to this sample, pressure wires and adenosine theoretically were not required in 21% of measurements yielding 100% accuracy with FFR as a reference standard. This analysis assumes that FFR is 100% accurate. The trade‐off for pressure‐wire–free procedures depending on aimed accuracy with FFR as a reference is illustrated in Figure S9.

### Subgroup Analysis

The precision (absolute difference of QFR‐FFR) for QFR with FFR as a reference was not different across strata of FFR values (0.06 for FFR, 0.55–0.64; 0.07 for FFR, 0.65–0.74; 0.04 for FFR, 0.75–0.84; and 0.04 for FFR, ≥0.85; *P*=0.47). Diagnostic accuracy was significantly reduced for lesions, with FFR 0.75 to 0.84 (was 100% for FFR 0.55–0.64; 92.7% for FFR 0.65–0.74; 71.3% for FFR 0.75–0.84; and 93.9% for FFR ≥0.85; *P*<0.001). We found no statistically significant difference in precision of QFR (absolute difference QFR‐FFR) per vessel (*P*=0.33) nor for tandem lesions versus single lesions (*P*=0.51). QFR was superior to 3‐dimensional quantitative coronary angiography diameter stenosis (AUC, 0.92 [95% CI, 0.89–0.95] versus 0.74 [95% CI, 0.69–0.80]; *P*<0.001) and 3‐dimensional quantitative coronary angiography area stenosis (AUC, 0.92 [95% CI, 0.89–0.95] versus 0.62 [95% CI, 0.55–0.69]; *P*<0.001; Figure S10). Diabetes mellitus showed statistical significant association with increased QFR‐FFR discrepancy (Table S6).

## Discussion

The FAVOR II E‐J study and the FAVOR II China study were the first multicenter studies investigating the feasibility and value of in‐procedure QFR calculated in the catheterization laboratory. The main findings of FAVOR II E‐J were: (1) The study confirmed the primary hypothesis with superior specificity and sensitivity of QFR compared with standard anatomical assessment by 2D‐QCA with FFR as a reference standard, and (2) QFR was feasible in a multicenter setting and was faster than FFR when analyzed during coronary angiography.

Diagnostic performance of QFR in FAVOR II E‐J was noteworthy and comparable with the findings in the recent and almost similar FAVOR II China study.^12^ SDs for mean difference FFR‐QFR were identical (0.06). The higher accuracy (92.7%) in FAVOR II China may be explained by the smaller number of lesions with FFR values close to the FFR 0.80 cutpoint. Results in both studies showed improved performance of QFR compared with early validation studies on offline computation of QFR.^11^, ^15^ The improved precision may be facilitated by the online analysis setup with instant feedback between operator and analyst. The standard operating procedure (Data S2), use of recommended angulations for angiographic projections (Table S3), and day‐to‐day feedback on enrolled cases may further have contributed to the improved results of QFR.

Most existing FFR computation methods for invasive coronary angiography predominantly rely on computational fluid dynamics.^5^, ^6^, ^16^ Inherited limitations of these methods may exist related to generating theoretical boundary conditions to create a “one‐size‐fits‐all”, and to long computation time for blood flow simulations. Morris et al recently presented a rapid computational fluid dynamics modality for calculation of virtual FFR with a high diagnostic precision (100% for FFR ≤0.80) and short mean time to virtual FFR (189 seconds).^17^ This study was performed using rotational angiography in a limited population of 20 patients. To our knowledge, the FAVOR II studies using QFR present the first data supporting that real‐time computation of FFR is feasible, fast, and accurate in patients with stable angina pectoris and applicable stenosis.

FFR is the established standard for invasive identification of flow limiting intermediate coronary lesions when no other objective evidence of lesion specific ischemia is present.^18^ The clinical adaption of FFR is increasing, but remains low.^1^, ^2^, ^19^ The underlying reasons may include the high cost of pressure wires, tortuous vessels, and the need for pharmacological hyperemia induction. Multiple studies presented approaches to avoid hyperemia for physiological lesion assessment, such as instantaneous wave‐free ratio and resting distal pressure/aortic pressure measurements. The resting indices perform similar with an overall diagnostic agreement between 80% and 90% when compared with FFR depending on distribution of lesions included in the studies.^20^, ^21^, ^22^, ^23^, ^24^ Still, instantaneous wave‐free ratio–based strategies versus an FFR strategy resulted in comparable clinical outcomes at 1 year in 2 large, randomized clinical trials.^23^, ^24^ We found a diagnostic accuracy for QFR (87%) comparable to the early instantaneous wave‐free ratio/FFR studies. Hence, the presented results support future comparison of FFR and QFR in clinical outcome trials.

Repeated core‐lab QFR analysis confirmed the agreement between QFR and FFR (identical SD of 0.06). However, direct comparison of in‐procedure QFR and core‐lab QFR revealed a small bias. The discrepancy indicates that the standard operating procedure for QFR computation might not have been sufficiently standardized for some lesion presentations or training was insufficient before study start. Core‐lab QFR showed less variation in disagreement at lower FFR values (Figure S5), indicating that contouring tight lesions could pose a specific challenge. Computation of QFR requires user interaction at steps, such as frame selection, lumen contouring, and contrast flow evaluation, and may hence be sensitive to small differences in the approach at various steps. A more elaborate standard operating procedure, more observer training, and automatizations are likely to reduce variation.

We showed that QFR is superior to standard quantitative coronary angiography in evaluating coronary artery stenosis. QFR may extend the access to physiology‐based guidance when access to pressure wires is limited by financial restrictions or inexpedient reimbursement systems. By enrolling patients where FFR is normally indicated, we included a distribution of lesions with a mean FFR approaching the clinical 0.80 cutpoint (mean FFR, 0.83±0.09). The vast majority of binary mismatches (treat/no‐treat) between QFR and FFR were cases close to the binary diagnostic cutoff, in whom the benefit of treatment approaches the percutaneous coronary intervention–related event rate.^25^ Although the study was not powered to do so, the sample allowed for the predefined assessment of a QFR‐FFR hybrid approach, which may reflect the true clinical application of QFR in centers with full adoption of physiology‐based diagnostics awaiting results of randomized outcome trials. Applying the 95% QFR‐hybrid limits (QFR‐treat 0.77 and QFR‐defer 0.86) to this population could potentially save pressure wires and adenosine in 64% of all lesions (Figure S8) and still ensure a diagnostic quality at the level of full FFR evaluation until clinical noninferiority of a QFR‐based diagnostic strategy has been established.

## Study Limitations

We only enrolled a limited portion of patients scheduled for secondary evaluation of coronary lesions after myocardial infarction. The diagnostic precision of QFR in nonculprit lesions, as recently assessed in a proof concept study by Spitaleri et al, could thus not be confirmed.^26^ We excluded lesions with Medina type 1.1.1 and 1.0.1 bifurcations attributed to specific limitations of the present QFR application; hence, the diagnostic precision of QFR in bifurcation needs further developments and investigation. Despite the inclusion of tandem lesions, we did not mandate FFR‐pullbacks during intravenous adenosine. Thus, a direct comparison between the FFR‐pullback curves and the spatially sensitive, color‐coded, continuous QFR values along the 3‐dimensional/angiographic roadmap could not be performed. Because FFR was the sole gold‐standard, we were not able to further characterize the lesion physiology in the presence of microvascular dysfunction. Time to QFR did not include the time for angiographic acquisition that could differ from an FFR‐based strategy. It is therefore not possible to determine whether use of the provided standard projections and requirement for limited overlap and foreshortening added procedure time. To emulate an integrated QFR solution, data transfer time from angiographic equipment to the QFR workstation was not included in time to QFR. In case of selection of a different view during analysis, the additional time was included in the time to QFR. Furthermore, preparing and zeroing the pressure system was not included in time to FFR because of site‐specific differences in the workflow.

## Conclusion

In‐procedure QFR is clinically feasible and is superior to angiographic assessment for evaluation of intermediary coronary artery stenosis when FFR is used as a reference. QFR bears the potential to expand the adoption of physiological lesion assessment.

## Sources of Funding

The study was funded by the Department of Cardiology, Aarhus University Hospital, Skejby and by the participating institutions. The manufacturer and distributor of the QFR software (Medis Medical Imaging bv., Leiden, NL) was not involved in design, conduct, or reporting of the study and provided no funding for the study except for making the Medis Suite solution available for free in the study period and provided training for participating sites.

## Disclosures

Westra received travel support and consultant fees from Medis Medical Imaging systems bv. Shengxian Tu received research from Medis Medical imaging systems bv. and Pulse medical imaging technology. Wijns received research grants (to his former institution) from stent manufacturing companies and speaker fees and honoraria from Biotronik, Mi‐Cell, and MicroPort. He is a co‐founder of Argonauts, an innovation facilitator. His research is supported by Science Foundation Ireland (Dublin). Holm received institutional research grants from Abbott, Boston Scientific, and Medis medical imaging. The remaining authors have no disclosures to report.

## Supporting information


**Data S1.** Supplemental Methods.
**Data S2.** FAVOR II Standard Operating Procedure for QFR Computation in FAVOR II Europe–Japan.
**Table S1.** Analysis Strategy
**Table S2.** Inclusion and Exclusion Criteria
**Table S3.** Recommended Angulations
**Table S4.** Number of Included Patients Per Site
**Table S5.** Diagnostic Accuracy
**Table S6.** Predictors of QFR‐FFR Discrepancy
**Figure S1.** Vessel‐level flow chart. FFR indicates fractional flow reserve; n, number of vessel; QCA, quantitative coronary angiography; QFR, quantitative flow ratio; RCA, right coronary artery.
**Figure S2.** FFR distribution. Disease severity according to fractional flow reserve (FFR). Mean FFR was 0.83±0.09 and 101 (32%) lesions were in the 0.75 to 0.85 interval. FFR indicates fractional flow reserve.
**Figure S3.** Per‐patient level diagnostic performance. Comparison of quantitative flow ratio (QFR) and 2‐dimensional quantitative coronary angiography (2D‐QCA) using FFR ≤0.80 as reference. AUC indicates area under the receiver curve; FFR, fractional flow reserve.
**Figure S4.** Per‐patient level correlation and agreement of QFR and FFR. Good per‐patient correlation (A) and agreement (B) of QFR and FFR was observed. Dashed lines in Bland–Altman plot illustrate mean difference ±2 SD. FFR indicates fractional flow reserve; QFR, quantitative flow ratio.
**Figure S5.** Core‐lab QFR correlation and agreement with FFR. Good correlation (A) and agreement (B) of between core‐lab QFR and FFR was observed. Dashed lines in Bland–Altman plot illustrate mean difference ±2 SDs. FFR indicates fractional flow reserve; QFR, quantitative flow ratio.
**Figure S6.** Per‐patient level diagnostic performance of core‐lab QFR and in‐procedure QFR. Comparison of in‐procedure quantitative flow ratio (QFR) and core‐lab QFR using FFR ≤0.80 as reference. AUC indicates area under the receiver curve; FFR, fractional flow reserve.
**Figure S7.** Correlation and agreement of core‐lab QFR and in‐procedure QFR. Good correlation (A) and agreement (B) of in‐procedure QFR and core‐lab QFR was observed. Dashed lines in Bland–Altman plot illustrate mean difference ±2 SDs. FFR indicates fractional flow reserve; QFR, quantitative flow ratio.
**Figure S8.** Clinical application of QFR. QFR limits to achieve ≥95% sensitivity (QFR‐treat 0.77) and ≥95% specificity (QFR‐defer 0.87) were identified for use in a QFR‐FFR hybrid approach. FFR indicates fractional flow reserve; QFR, quantitative flow ratio.
**Figure S9.** QFR‐FFR hybrid strategy. The diagnostic agreement between FFR and QFR increases with adjusted QFR‐treat and QFR‐defer limits. With increasing diagnostic agreement, fewer lesions are evaluated without pressure‐wires and adenosine. This analysis assumes that FFR is 100% accurate. FFR indicates fractional flow reserve; QFR, quantitative flow ratio.
**Figure S10.** Per‐vessel level diagnostic performance of in‐procedure QFR, 3D‐QCA, and 2D‐QCA. Comparison of in‐procedure quantitative flow ratio (QFR), 2D‐QCA, and 3D‐QCA using FFR ≤0.80 as reference. % DS indicates percent diameter stenosis; % AS, percent area stenosis; 2D‐QCA, 2‐dimensional quantitative coronary angiography; 3D‐QCA, 3‐dimensional quantitative coronary angiography; AUC, area under the receiver curve.
