# Supplementary material for: Diagnostic Performance of In‐Procedure Angiography‐Derived Quantitative Flow Reserve Compared to Pressure‐Derived Fractional Flow Reserve: The FAVOR II Europe‐Japan Study
Source: J Am Heart Assoc. 2018 Jul 6;7(14):e009603. doi: 10.1161/JAHA.118.009603 (PMC6064860; doi:10.1161/JAHA.118.009603)
Supplement: Supplementary file 1 — Data S1. Supplemental Methods. Data S2. FAVOR II Standard Operating Procedure for QFR Computation in FAVOR II Europe–Japan. Table S1. Analysis Strategy Table S2. Inclusion and Exclusion Criteria Table S3. Recommended Angulations Table S4. Number of Included Patients Per Site Table S5. Diagnostic Accuracy Table S6. Predictors of QFR‐FFR Discrepancy Figure S1. Vessel‐level flow chart. FFR indicates fractional flow reserve; n, number of vessel; QCA, quantitative coronary angiography; QFR, quantitative flow ratio; RCA, right coronary artery. Figure S2. FFR distribution. Disease severity according to fractional flow reserve (FFR). Mean FFR was 0.83±0.09 and 101 (32%) lesions were in the 0.75 to 0.85 interval. FFR indicates fractional flow reserve. Figure S3. Per‐patient level diagnostic performance. Comparison of quantitative flow ratio (QFR) and 2‐dimensional quantitative coronary angiography (2D‐QCA) using FFR ≤0.80 as reference. AUC indicates area under the receiver curve; FFR, fractional flow reserve. Figure S4. Per‐patient level correlation and agreement of QFR and FFR. Good per‐patient correlation (A) and agreement (B) of QFR and FFR was observed. Dashed lines in Bland–Altman plot illustrate mean difference ±2 SD. FFR indicates fractional flow reserve; QFR, quantitative flow ratio. Figure S5. Core‐lab QFR correlation and agreement with FFR. Good correlation (A) and agreement (B) of between core‐lab QFR and FFR was observed. Dashed lines in Bland–Altman plot illustrate mean difference ±2 SDs. FFR indicates fractional flow reserve; QFR, quantitative flow ratio. Figure S6. Per‐patient level diagnostic performance of core‐lab QFR and in‐procedure QFR. Comparison of in‐procedure quantitative flow ratio (QFR) and core‐lab QFR using FFR ≤0.80 as reference. AUC indicates area under the receiver curve; FFR, fractional flow reserve. Figure S7. Correlation and agreement of core‐lab QFR and in‐procedure QFR. Good correlation (A) and agreement (B) of in‐procedure QFR and core‐l [file JAH3-7-e009603-s001.pdf]

SUPPLEMENTAL MATERIAL

Data S1.

Supplemental Methods

Sample size calculation

Estimates for the sample size calculation were based on the results from the FAVOR study, where a sensitivity of 0.74 and a specificity of 0.91 for QFR were found. The null hypothesis was H0: Sensitivity(QFR≤0.80) = Sensitivity(%DS≤50) and H1: Unequal sensitivities for the two methods. A normal-approximate McNemar test with the Connor method was performed; Proportion1=0.48; proportion2=0.74; correlation=-0.1. We did similar for specificity (H0: Specificity(QFR>0.80) = Specificity(DS>50), and H1: Unequal specificities for the two methods; Proportion1=0.75; proportion2=0.91; correlation=0.4. With power=0.90, alpha=0.05 and a rate of true positives in the population of estimated 30%, a total of 274 patients with paired QFR and FFR were required to reject the null hypothesis for sensitivity and 257 for specificity. To accommodate for insufficient angiographic quality or failed FFR a total of 310 patients were estimated to be required.

Secondary endpoints

Feasibility

The feasibility was assessed as the fraction of lesions with successful FFR measurements where QFR was computed.

Time to QFR and FFR

Time to QFR was defined as start of frame selection for the three-dimensional reconstruction of the vessel until QFR was computed using contrast flow evaluation. Time to FFR was defined as the

introduction of the pressure wire to the guiding catheter until drift check with a drift value within the specified limits.

QFR/FFR hybrid-approach limits

For a QFR/FFR hybrid strategy we used an FFR-only strategy as gold standard. QFR limits to yield a sensitivity (QFR-treat) and specificity (QFR-defer) of 90 and 95 percent were identified and used to model a hybrid approach where wire-based FFR assesment is needed between the QFR-treat and QFR-defer limits. The proportion of potential pressure wire free lesion assessments was calculated.

Prediction of QFR-FFR discrepancy

We constructed a multilevel mixed effect model including sites as level variable. Following co-variates were tested individually and included in the multivariate analysis if P-value<0.10: lesion length, % DS (2D-QCA), age, BMI, adenosine route, sex, smoking, vessel, diabetes, previous PCI, and FFR.

Procedure training

Participating sites were requested to have operators and dedicated staff trained on QFR computation. The staff received instructions and training from Medis medical imaging bv. Only staff with QFR certificates obtained from Medis could perform the study computation of QFR. Besides the QFR training from Medis, all sites were required to submit at least two complete and fully anonymized training datasets for approval by the respective core-labs before study enrolment.

Site specific blinding protocol

Blinding was ensured by one of the following site-specific strategies: 1) QFR was computed simultaneously in a separate room (Skejby, Naples, Ferrara, Warsaw, Gifu, Madrid, Essen, Mestre) 2) QFR was performed before FFR (Caserta, Skejby, Hague).

Data S2.

FAVOR II standard operating procedure for QFR computation in FAVOR II Europe-Japan

The QFR standard operating procedure (SOP) applied by all sites in the FAVOR II multicenter study by Aarhus University Hospital, Skejby, Denmark. The set of instructions do not constitute a manual, neither partly nor in full, for clinical use of QFR.

1. Identification of cases not appropriate for QFR during coronary angiography (angiographic exclusion criteria)

1.1 Aorto-ostial stenosis

Aorto-ostial stenosis is not analyzable by QFR at present due to the requirement of two optimal projections, the guiding catheter intrusion and back flow of contrast in aorta overlapping the ostium.

1.2 Low angiographic quality or poor contrast filling

In some cases, the application is not able to recognize the vessel contours due to excessively low angiographic quality or poor contrast filling and exclusion of the case can be necessary (fig. 1). With experience the operator may decide to exclude the case even before transmitting runs to the QFR work station for analysis.

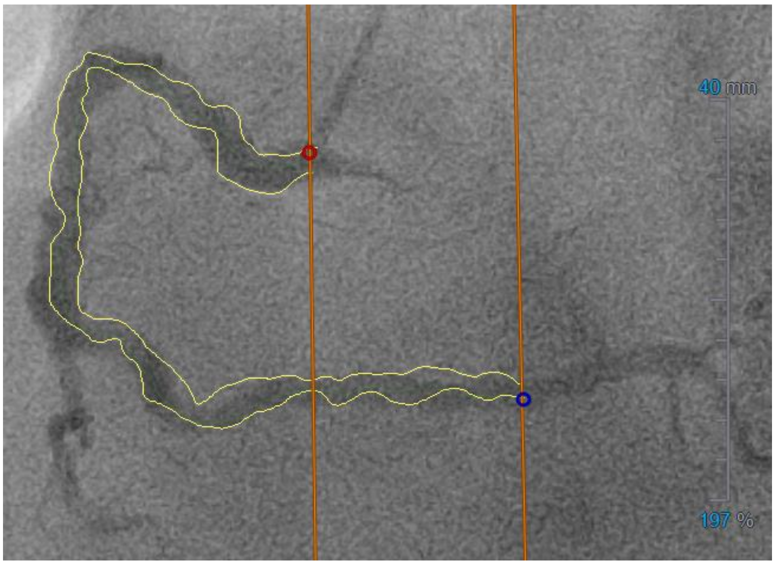

__Figure 1__ Low angiographic quality. The QFR application has difficulties finding the vessel lumen and vessel borders.

1.3 Overlap

If correct lumen contouring is impossible due to severe overlap of the stenosed segment, the case should be excluded.

1.4 Nitroglycerin administration

When nitroglycerin is not administered neither systemic nor intracoronary, vessel spasms cannot be ruled out, and the case should be excluded (fig. 2). Without prior nitroglycerin, both the QFR analysis and FFR measurement can be unreliable.

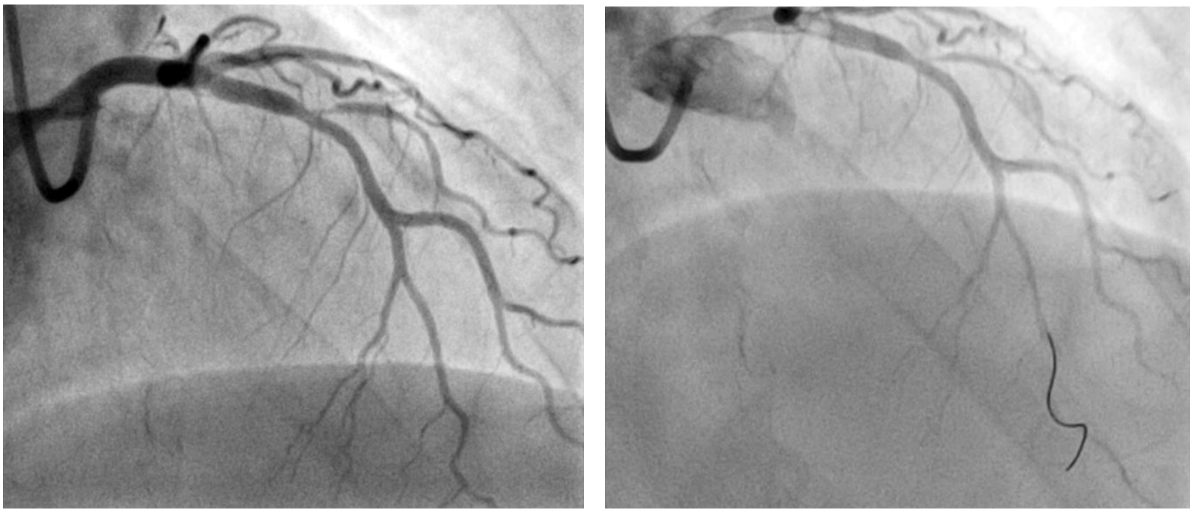

Figure 2 *FFR measurement without nitroglycerin before and after advancement of wire. The wire causes the vessel to spasm and therefore the FFR value is unreliable.*

1.5 Stenosis at or near large diameter shifts

QFR validity is unknown in bifurcation lesions if the stenosis involves both sides of a major shift (>1 mm) in reference diameter. This includes:

- Patients with lesions in the distal LMCA and the ostium of the Cx.

1.6 Severe tortuosity of target vessel

Severely tortuous vessels where excess foreshortening of the stenosed segments cannot avoided should not be analyzed by QFR.

2. Step-by-step manual

The Medis Suite QAngio XA 3D/QFR solution (Medis medical imaging system bv, Leiden, The Netherlands) is used for computation of QFR in FAVOR II. The Medis Suite QAngio XA 3D/QFR solution requires installation on a Windows-based computer. QFR computation is described step-by-step below.

2.1 Coronary angiography

Two good projections at least 25 degrees apart are required for the 3D vessel reconstruction. Angiographic procedure:

- Inject I.C. nitroglycerin as early as possible
- Use framerate of at least 12.5 frames/sec
- Make sure that the catheter is filled with contrast before the injection (i.e. after administration of nitroglycerin)
- Use brisk, continuous and fast contrast injections. Aim for filling during full 3 cardiac cycles
- Minimize overlap of target segments
- Avoid foreshortening of the vessel
- Avoid zooming but use of other means to increase image quality are encouraged.
- Avoid moving the table early after injection
- Aim for projections perpendicular to the target vessel – consider suggested projections (table1)
- Make sure that the entire vessel is visible in both projections. Both the guiding catheter tip and the potential position of the FFR pressure transducer should be visible in the same frame

Suggested projections are found in table 1.

Table 1 *Recommended projection angles for specific lesion segments. Angulation of more than 25° between projections is required.*

Vessel /Bifurcation	1st View	2nd View
LM + LAD/LCX	RAO 20, Caudal 45	AP, Caudal 10
LAD/Diag	AP, Cranial 45	RAO 35, Cranial 20
LCX/OM	LAO 10, Caudal 25	RAO 25, Caudal 25
Proximal+Mid RCA	LAO 45, CAUD 0	AP, CAUD 0
PLA/PDA	LAO 45, CAUD 0	LAO 30, CAUD 30

If only one good projection is identified, consider to use the *Acquisition Guide* in the Medis Suite QAngio XA 3D/QFR solution to identify the second projection:

1. Transfer first good projection to QFR computer (see 2.2)
2. Right-click on the projection and start the QAngio XA 3D application
3. Choose *Acquisition Guide* (*fig.3*, red box). The yellow line indicates the new projection angle, and should be approximately perpendicular to the target vessel at the lesion site

    a. If several lesions are located in the same vessel, a compromise must be made to ensure that most of the lesions and the most severe lesions are seen in the same projection

4. Move the projection line by moving the yellow spot (fig.3, white arrow) in the Acquisition Guide indicator. Aim to keep the yellow spot inside the green area and to achieve an angle difference of 30-50 degrees
5. Position the C-arm as proposed by the guide
6. In case of excessive overlap of the target segments and other vessels, rotate the C-arm 5 degrees around the axis of the target vessel
    a. If needed, use the Acquisition guide indicator again by maintaining the angulation of the yellow line and move the yellow spot just outside the green area – away from the red area. Move the C-arm accordingly to the new proposition

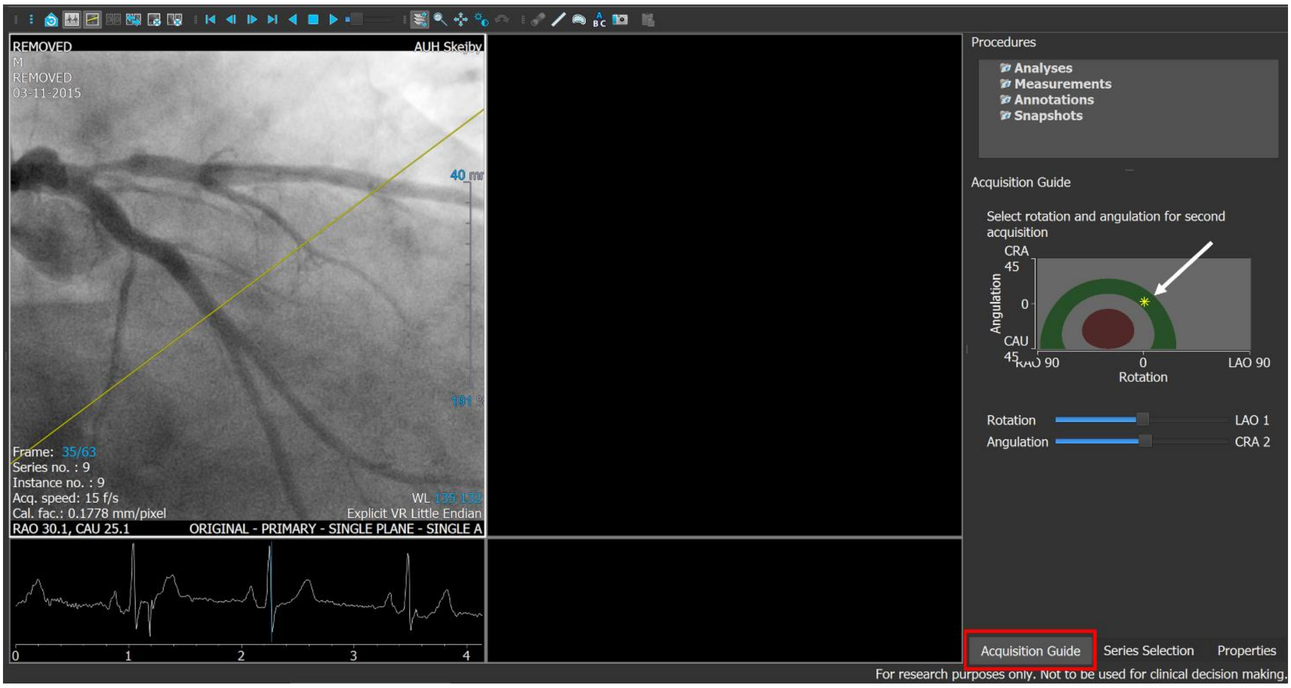

***Figure* 3** *Acquisition projection angle. Red box: Acquisition guide. White arrow: Yellow spot indicating position of C-arm.*

2.2 Image transfer

The angiographic runs are transferred to the QFR-computer using an angiographic equipment specific protocol.

2.3 Angiographic run selection

Optimal projections are chosen according to the following criteria:

- Minimal overlap of the target vessel
- Good contrast injection, filling the entire vessel
- Includes both the healthy part of the vessel proximal to the first stenosis and the location of the pressure transducer of the subsequent FFR assessment

Workflow in angiographic run selection

1. Identify the optimal projection and right-click the best run. Start QAngio XA 3D
2. Choose *Series Selection* (fig.4, red box) to get a presentation of angiographic runs that are ≥ 25 degrees different from the selected run
3. Evaluate the potential runs by dragging them into the empty, right panel (fig.4*)*
    - If the two projections are not 25 degrees apart, you can change the minimum angle in the pop-up menu in Options. It is not recommended to do the 3D reconstruction based on runs <25 degrees apart
4. Keep the best 2nd run in the panel with the epipolar line perpendicular to the lesion(s)

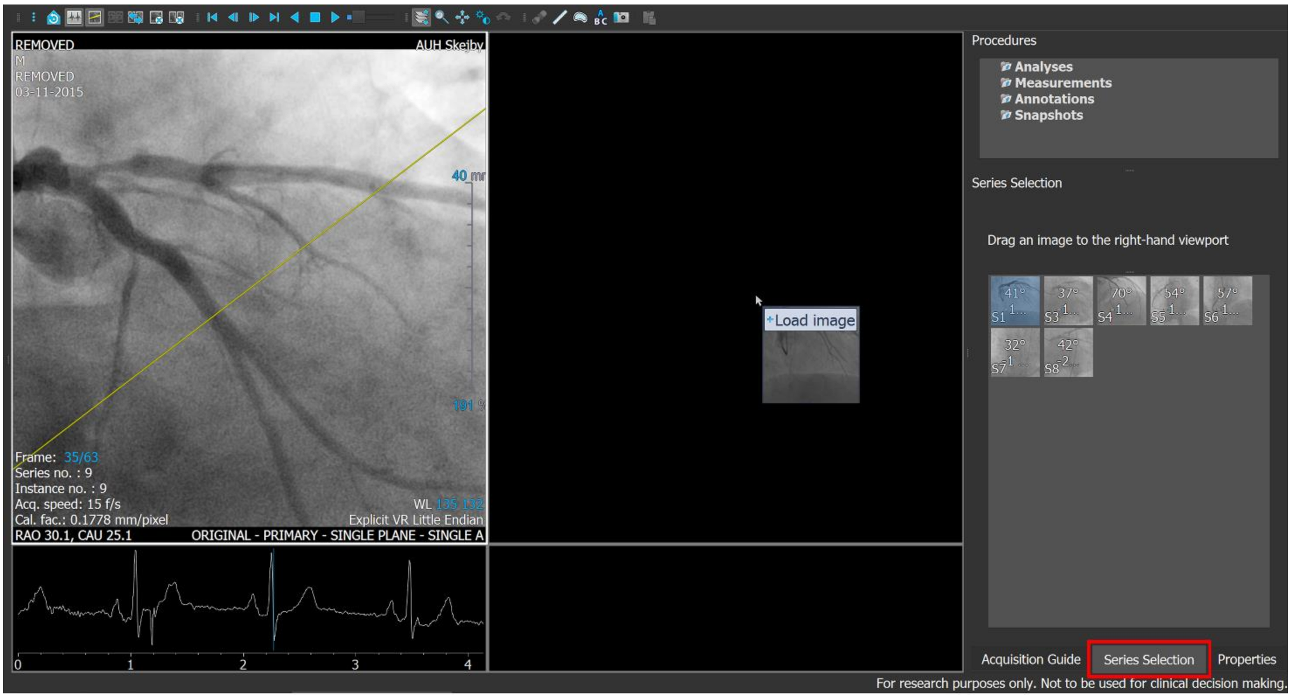

***Figure 4** Angiographic run selection. Red box: Series Selection.*

2.4 **Frame selection**

The best frames for analysis are selected by ensuring:

- The lesion site(s) is not overlapped
- The entire vessel is filled with contrast
- Frame includes both the healthy part of the vessel proximal to the first stenosis and the location of the pressure transducer of the subsequent FFR assessment
- Frames are ”end-diastolic” – preferably frames recorded between the P-wave and the QRS-complex (fig.5)

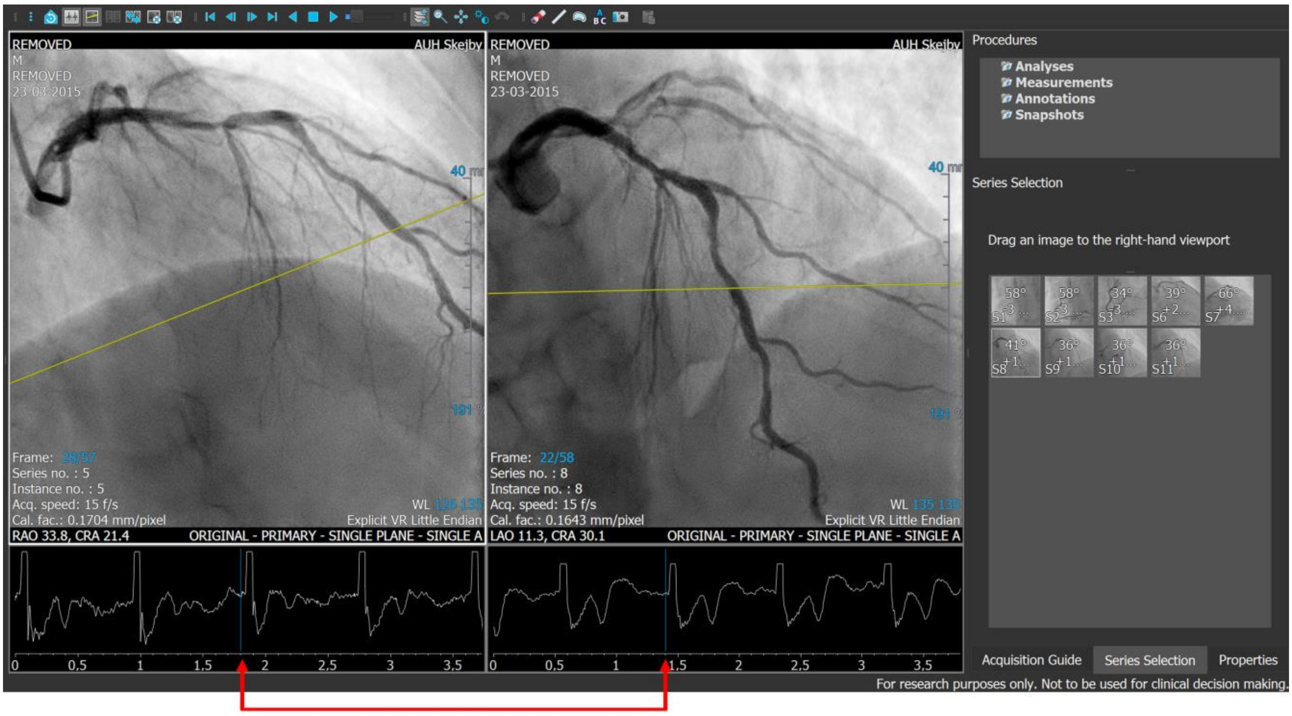

Figure 5 *Frame selection. Note that both runs are in the same end-diastolic phase and the epipolar line is perpendicular to the lesions.*

When the best end diastolic frame is found in both panels, the 3D reconstruction is initiated by pressing the *Create single vessel analysis*-button (fig. 6, red arrow) in the top panel.

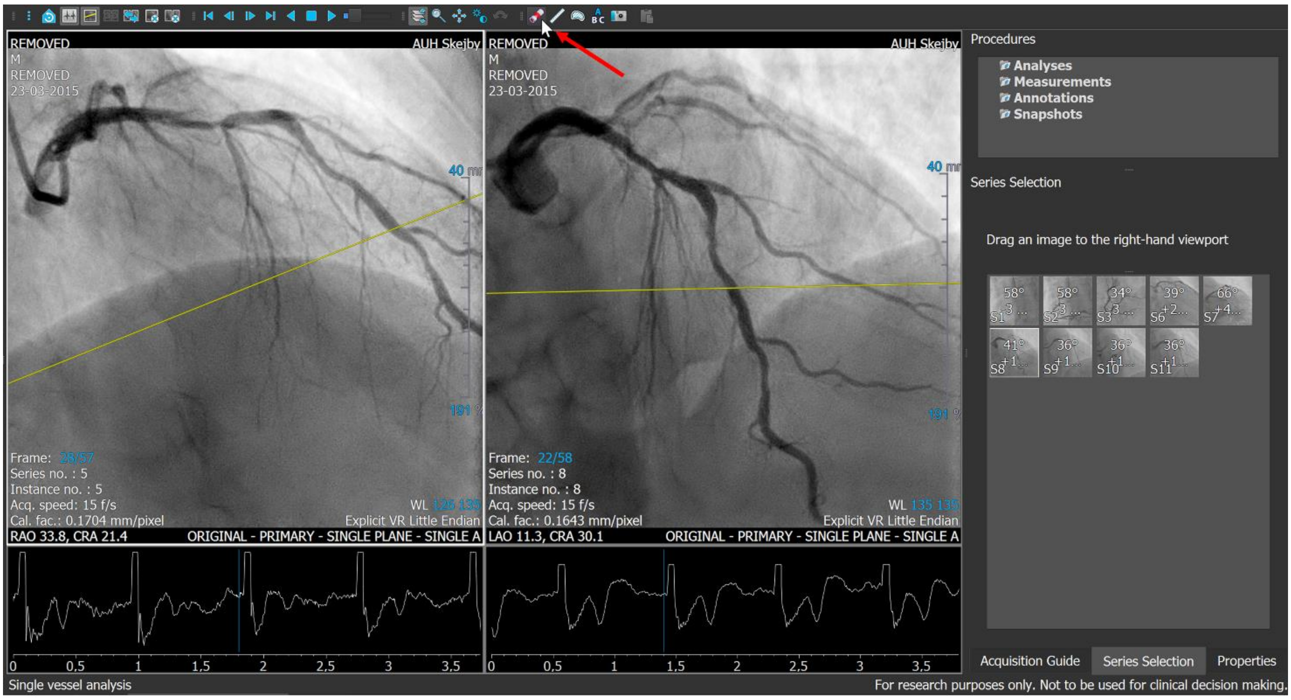

Figure 6 *Create single vessel analysis (marked by red arrow) initiates the 3D reconstruction.*

2.5 3D target vessel reconstruction

To link the two projections, corresponding landmarks near the lesion are identified by a pair of offset points in both projections (fig. 7). Make sure to:

- Identify a landmark that is easily identified in both projections (i.e. a bifurcation, a localized stenosis or the off-spring of a side branch)
- If using a side branch:
    - Select a side branch that departs perpendicularly from the main branch - if possible
    - Place the offset point in the middle of the main vessel

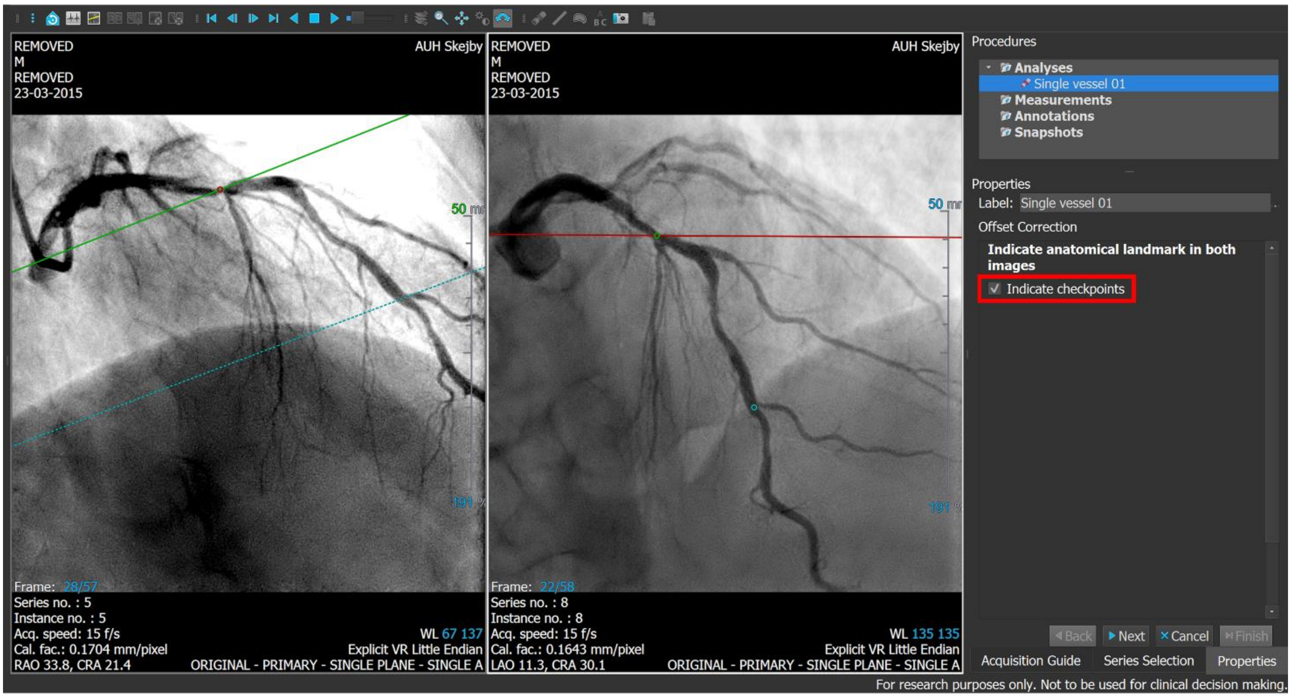

Figure 7 *Corresponding point is marked by the red and green spot in the left and right panel, respectively. In this example, the offspring of a side branch/lesion point is easily recognized in both projections, is selected as the point to correspond.*

- Use the *Indicate checkpoints* option, to make sure that the projections are linked together properly.
  - Tick off the *Indicate checkpoints* box (fig. 7, red box)
  - Choose another landmark, identifiable in both projections (i.e. a bifurcation, a stenosis or the off-spring of a side branch)
  - Put a checkpoint proximal and distal to the corresponding point or place another checkpoint to check the agreement for reconstruction between the two projections

The matching checkpoints are shown as a circle in one projection and as a dotted line in the other, in the same colour.

- Revise the position of the chosen offset point or select an entirely different location for the offset point if the checkpoints are not consistent in the two projections

2.6 Indicating target vessel

Indication of the boarder of segment to analyse

1) Ensure that the analysed segment includes reference segments at both ends for optimal reference vessel reconstruction
2) The proximal path line point is placed in a “most healthy” part of the vessel, proximal to all stenotic segments
3) When the proximal path line point is added in one panel, a corresponding support line is shown in the other panel. The proximal path line point in the second projection is placed on this support line at the same anatomical location
   a. If the corresponding support line is parallel to the proximal or distal part of the vessel it can be necessary to place the proximal point in relation to an anatomical landmark that can be recognized in both projections
   b. If the proximal parts of the vessel corresponds poorly, the proximal point in the second projection should not be placed at the indicator line, but landmarks should be used to ensure the same position of the proximal points in the two projections-Later, the projections may need to be “*forced corresponded*” (see 2.9).
4) The distal point is placed at least as far down as the pressure transducer is positioned during the FFR measurement

After indicating proximal and distal points in both projections, the vessel pathline (fig. 8) is shown. The path line is verified visually for both projections. If it deviates from the target vessel, it is dragged into position using support points.

When the position of the vessel pathline is accepted, the pathline is “locked” (fig. 8, red box).

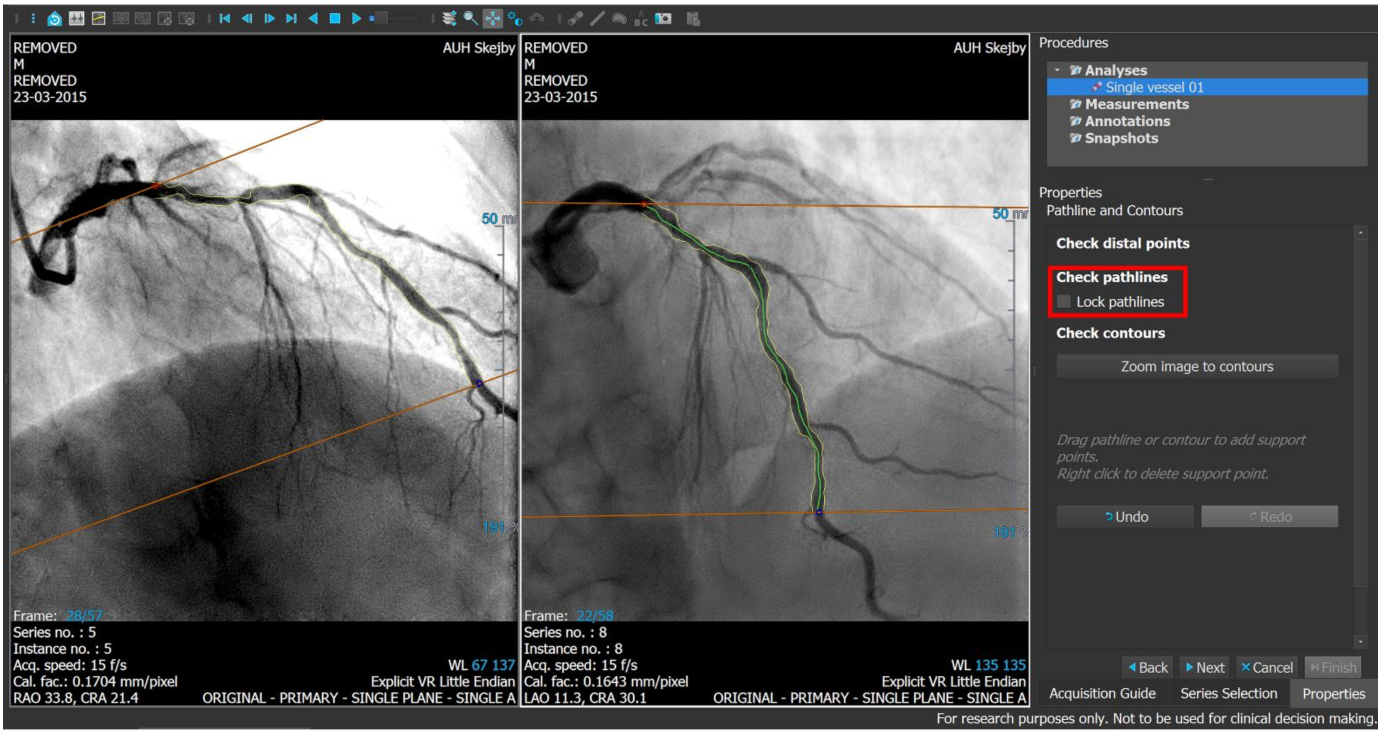

***Figure* 8** *Segmented target vessel. The proximal point is marked by red circles while the distal point is marked by blue circles. The target vessel pathline is indicated by the green line in the right panel. It is visible when the mouse is shifted over it. The pathline is fixated by ticking Lock pathlines ‘(red box).*

2.7 Lumen contouring

The yellow contour lines (fig. 9) are adjusted to follow the lumen border. **Pay special attention to:**

- Indication of non-existing narrowings in the proximal and distal ends
- Correct contouring of the target lesion(s)
- Side branches and overlap
- Ensure that contours are correct in all segments – also non-target segments as it influences QFR calculation

The lines are corrected by dragging them into position with correction points. If a correction needs to be reverted, right-click the created correction-point and it will be deleted.

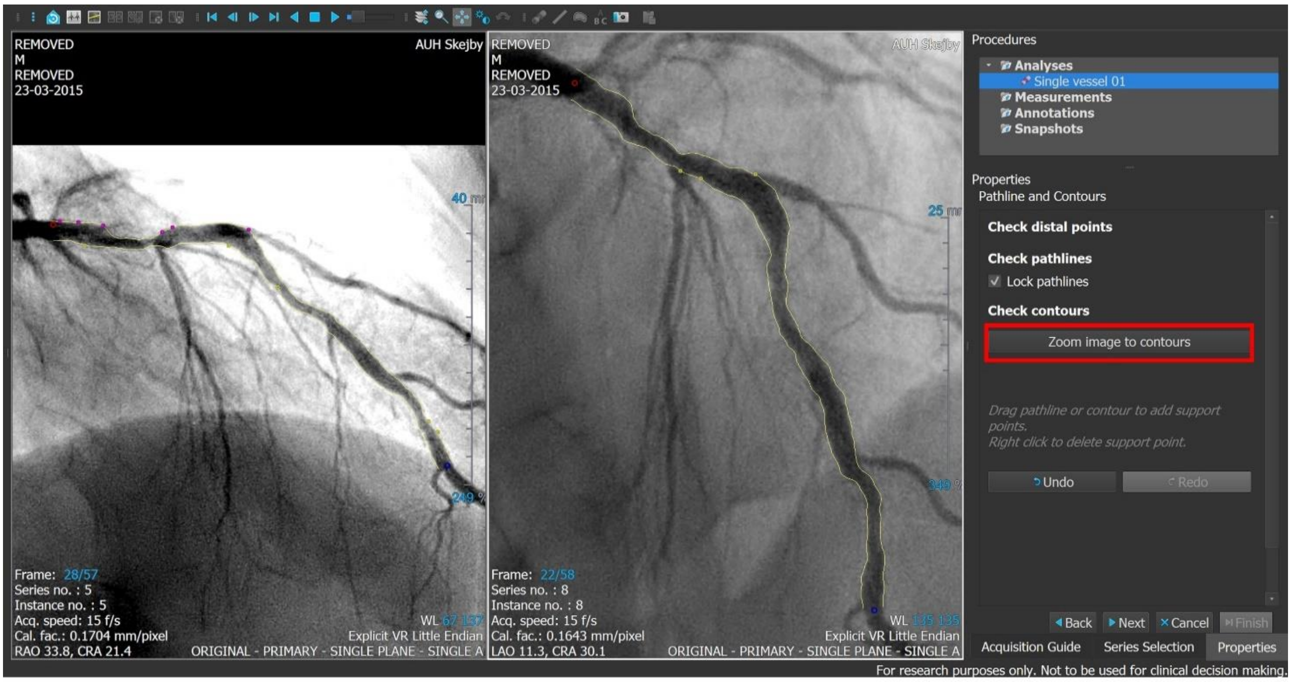

Figure 9 *Lumen contouring. The yellow lines indicate the lumen border, and can be corrected by dragging them into position (note the placed correction points). To get a better view, click "zoom image to contours (red box).*

2.8 **Forced correspondence**

Correspondence is automatically performed, but manual forced corresponding points can be added for editing. With this tool, the two centerlines are forced to correspond at an indicated point. Forced correspondence is particularly important when the graphs for the two minimal and maximal diameters in lower right panel are shifted sideways instead of being almost superimposed (fig. 10).

1) Identify an anatomical landmark easily identified in both projections (i.e. the narrowest part of the target lesion or the off-spring of a side branch)

Tick Corresponding points

1) Indicate the landmark in both projections
2) Check if the curves of maximum and minimum diameters are now more aligned
3) Adjust the markers until finding the best possible correspondence, with good alignment of the two curves

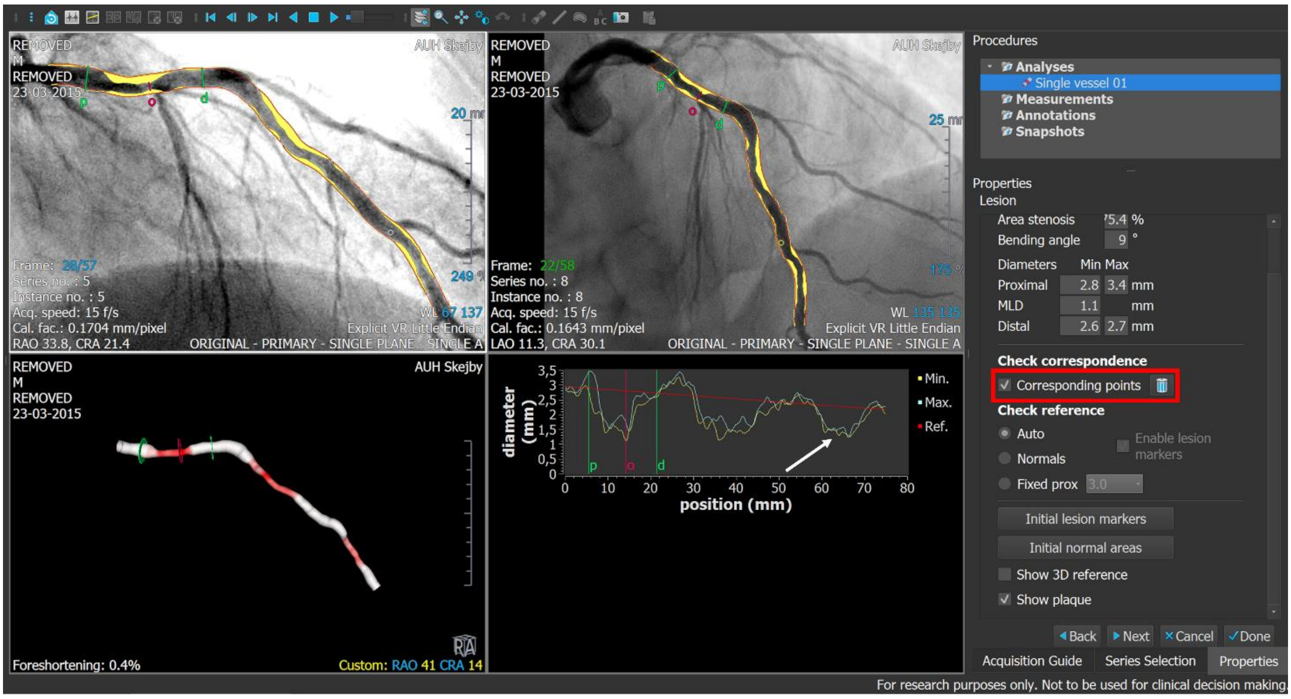

Figure 10 *Forced corresponding points. The option is marked (red box) and landmarks are selected (see text). Note the improved distal alignment between the two diameter functions (blue and yellow lines. White arrow) compared to figure 10-12.*

NOTE: Focus on getting the lesions and proximal vessel segments to correspond. Use the lesions markers to check the correspondence at this step before proceeding.

2.9 Reference vessel

Every part of the contoured lumen that is narrower than the reference vessel is marked yellow as plaque (fig. 11). These yellow markings can be removed by ticking off the box *Show plaque* (Figure 11, red box).

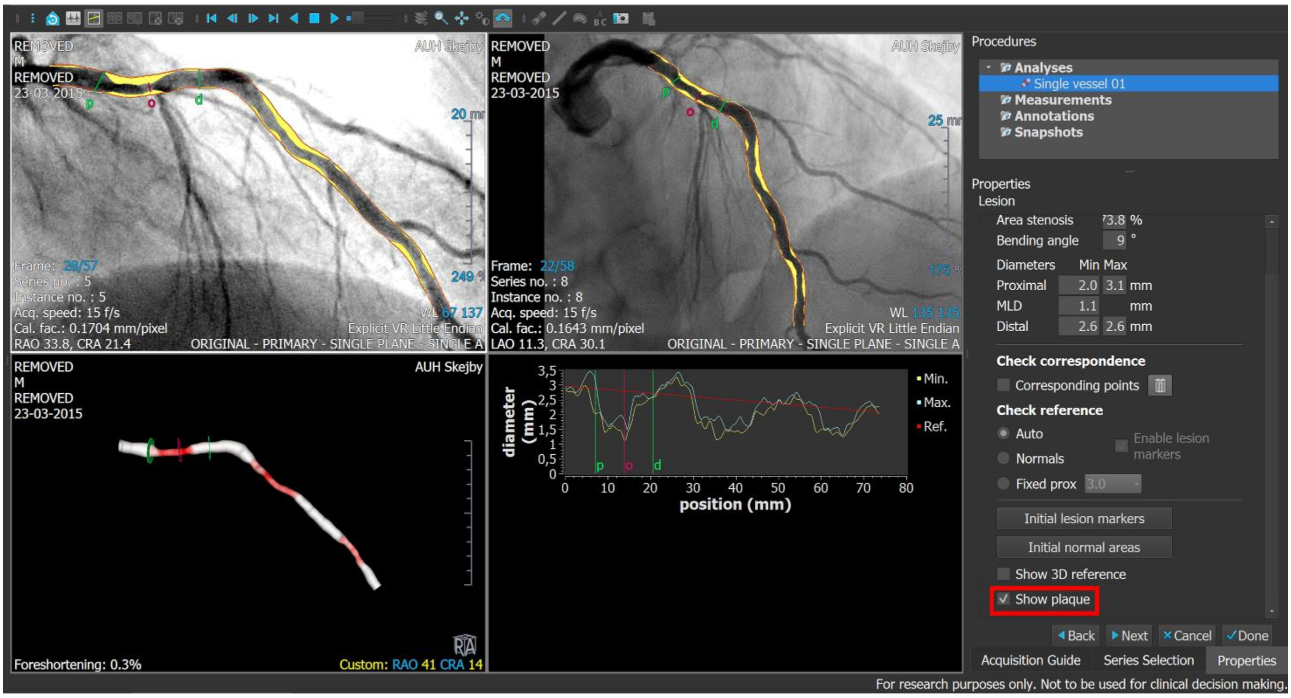

***Figure 11** Reference vessel (red contours on 2D images). Show/hide yellow plaque (red box).*

The proximal and distal ends are supposed to approximately match the healthy vessel parts. The reference function should obey the following:

- Always tapering reference function. A straight function is allowed in short segments.
- Should not follow stenosed or aneurysmatic sections.
- Sizes should be realistic according to gender and body mass index. See 2.9.3

If the abovementioned criteria are not fulfilled, the reference contours can be edited as follows:

2.9.1 Selection of normal areas

Select “Normals” under the “check reference wizard” (text fig. 12, red box). Select two normal areas, using the green areas. The reference function is now calculated as a linear regression based on the two selected normals. Note the slightly adjusted reference contours after using the “Normals” function (fig. 12 compared to fig. 11).

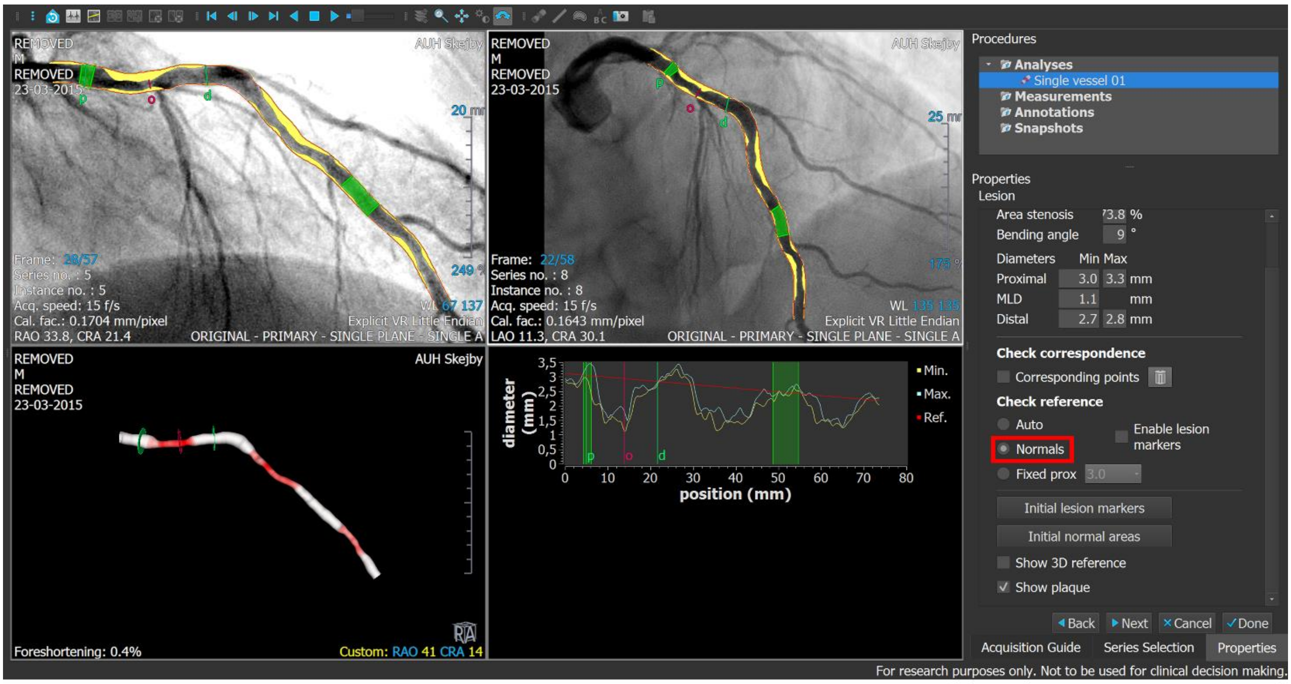

__Figure 12__ Reference function editing using the ”Normals” function (red box). The green normal areas are selected to indicate two healthy vessel segments.

2.9.2 Fixed proximal reference

To impute a reference size for a particular segment use the fixed reference tool. Select “Fixed prox” under the “Check reference” (text fig 13, red box). A fixed proximal reference size is selected with a 0.25 mm interval from 2 to 5 mm. Place the proximal green marker where the vessel should

have the indicated value. A normal distal area is chosen to adjust the slope of the linear function (see fig. 13).

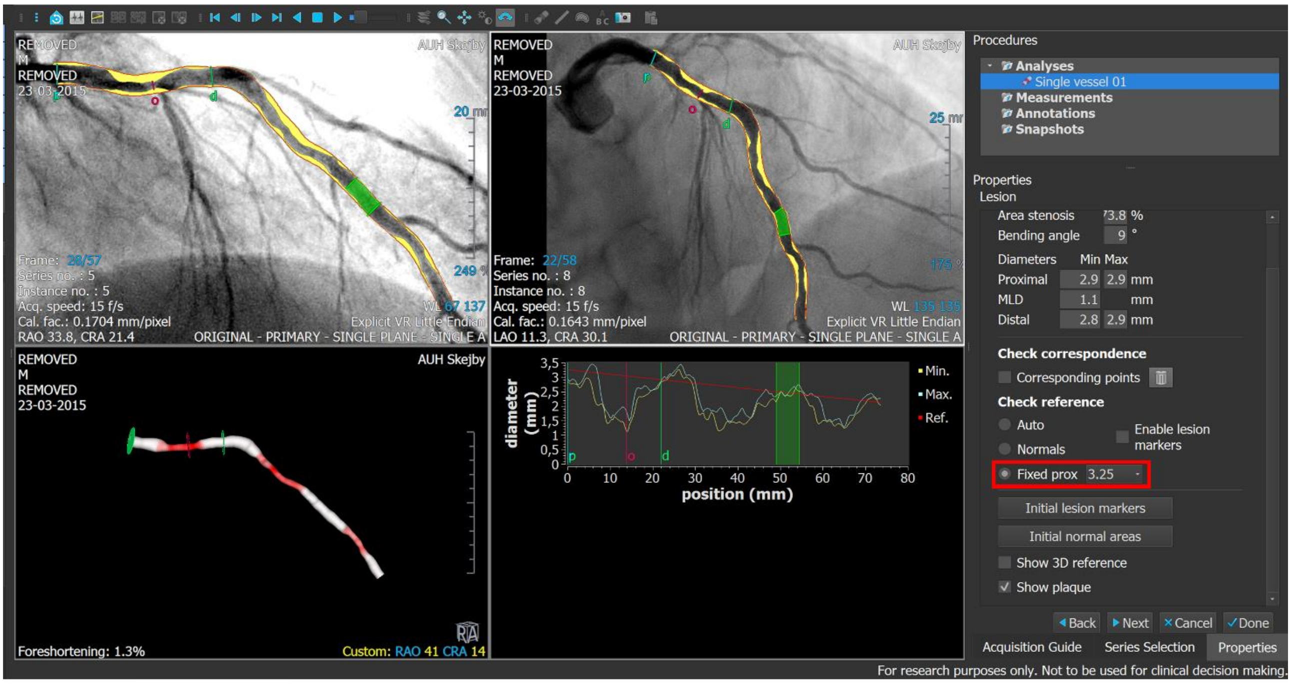

Figure 13 *Reference function editing using the "Fixed Prox" function (red box). The normal areas is moved to indicate a healthy distal vessel segment.*

2.9.3 **Reference diameter strategy**

If the automatic generated reference function based on the 3D-recontruction follows the criteria (2.9), it is used as the first choice. If not satisfied, selection of normal areas (2.9.1) is recommended in vessels with:

- Clearly identifiable heathy segments
- Realistic proximal reference size of the vessel according to gender and body mass index

A fixed proximal reference (2.9.2) is recommended in cases with:

- Proximal LAD disease defined as a proximal LAD reference size < 2.5 mm for women and < 3.0 mm for men in Caucasians with healthy segments distally
- Diffuse LAD disease with segments in mid/distal vessel parts exceeding the proximal reference size

The fixed proximal diameter is set to 3.0-3.5 mm men and 2.5-3.0 mm for women depending on the size of LM and LCx) and the patient (age, MBI).

Verify the reference diameter (criteria from 2.9) by looking at the diameter graph in the lower right panel. The red line indicates the reference lumen diameter, and the two graphs the minimum and maximum lumen diameters from the two images.

NOTE: it is more **important to have a correct reference function by manual adjustments** than preserving a wrongly automatic generated reference function to aim for reduced variability

2.10 **Fixed flow QFR computation**

1. Press next
2. Indicate Nitro yes or no
3. Enter *Vessel segment*: Left main/LAD or Other coronary (fig 14, red box)

   A Fixed Flow QFR will now be calculated

2.11 Frame count based QFR computation

For a potentially more accurate calculation of QFR, frame count based computation is performed;

1. Frame count (Figure 14, yellow box)

a. Choose projection for frame count, either

    i. The left panel run

    ii. The right panel run

    iii. Another projection

    The projection in which frames are counted should show good contrast filling, have a constant contrast flow/speed, a frame rate of at least 12.5 frames/sec for contrast QFR and 25 frames/sec for adenosine QFR.

b. *Start frame* is indicated as the frame in which the contrast arrives at the proximal pathline point

c. *End frame* is indicated as the frame in which the contrast arrives at the distal pathline point

d. If the proximal or distal point pathline is reached by the contrast between two frames, the first of the two frames is chosen, and the *+1/2*-box is ticked off

e. Another option is to relocate the proximal and the distal vessel delimiters, to get a better correspondence between the chosen start or end frame and the contrast position

PLEASE NOTE that projections where the contrast seems to appear uniformly in most of the analysed segment simultaneously are not appropriate for frame count. Remember to enter patient state (the angiographic run for frame counting is acquired during resting condition or hyperaemia).

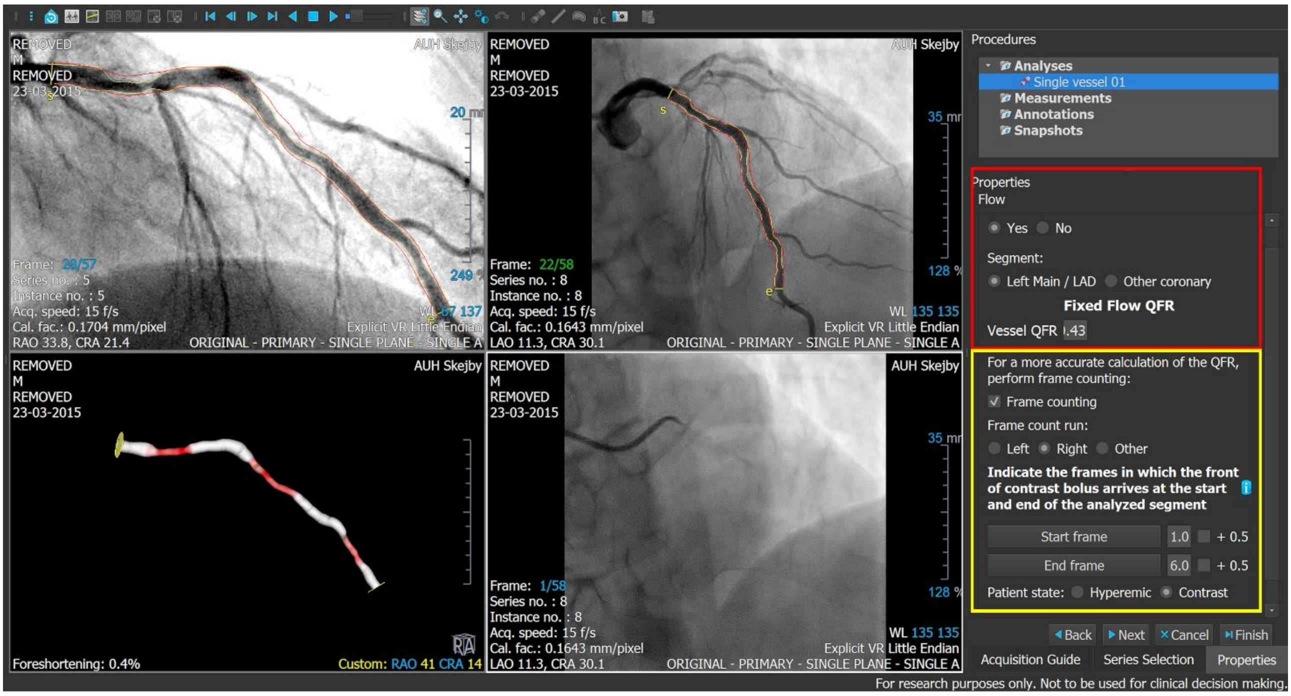

Figure 14 *Red box: indicate vessel for fixed flow QFR computation. Yellow box: Frame count QFR analysis by indicating the start- and end frame for contrast flow through the segmented vessel part. Frames are found by scrolling through the selected run in lower right image panel.*

After frame count QFR is calculated the following QFR-values are listed (fig 15):

- Lesion QFR: calculated for the lesion segment between the two green lesions markers. Segments proximal to the proximal lesion marker are considered non-stenotic
- Vessel QFR: calculated for the entire contoured segment. Segments proximal to the contoured segment are considered non-stenotic
- Index QFR: calculated from the proximal end of the contoured segment to the user defined white index line. The index line can be moved within the contoured vessel segment. When comparing QFR and FFR directly, make sure to place the index line at the site of the

pressure transducer on the pressure wire. To identify the wire position, choose ‘View’-state and identify the wire position.

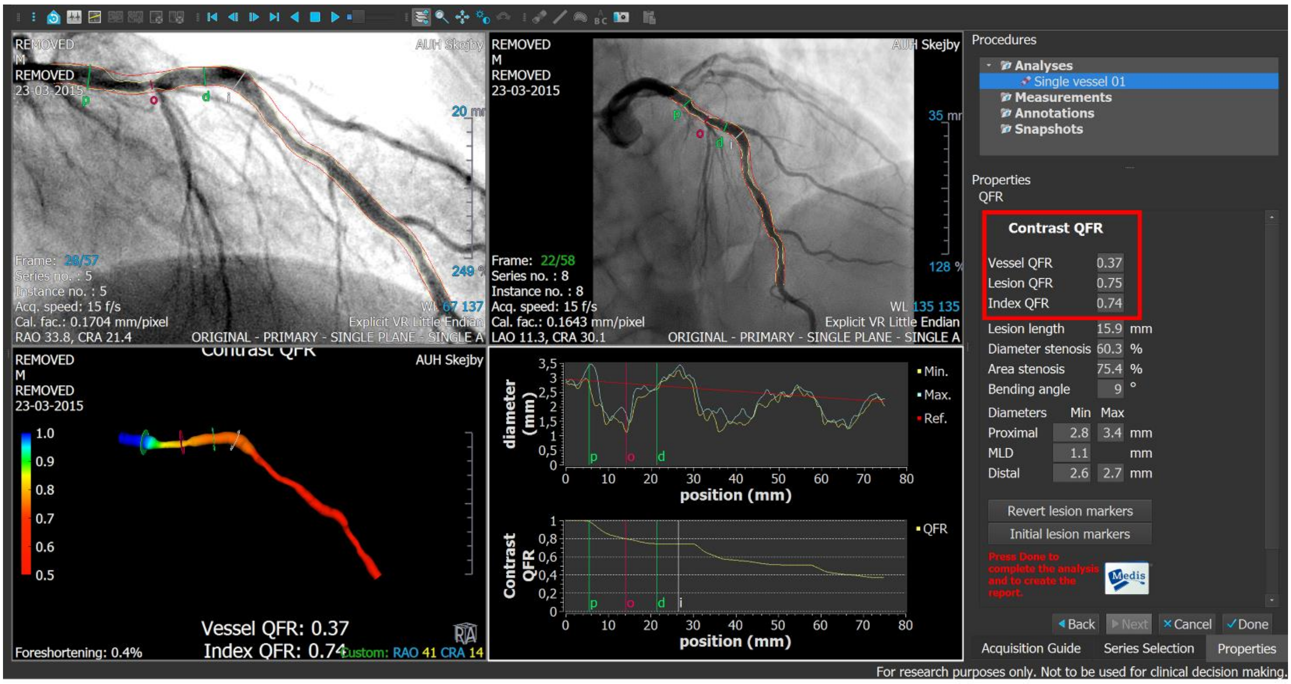

Figure 15 QFR results (red box).

2.12 Documentation

After finalizing analysis, it is saved by two steps for the study purpose.

1. A screenshot is acquired and saved in a folder named ”Patient X”, X indicating the patient’s study ID. The screenshot should be of the entire screen including the time and date in lower right corner of Windows
2. The QFR analysis is saved in the Medis Suite QAngio XA 3D/QFR solution by clicking *Done* in the lower right corner and clicking save as in the upper panel to save with study ID (Figure 16, red marker)

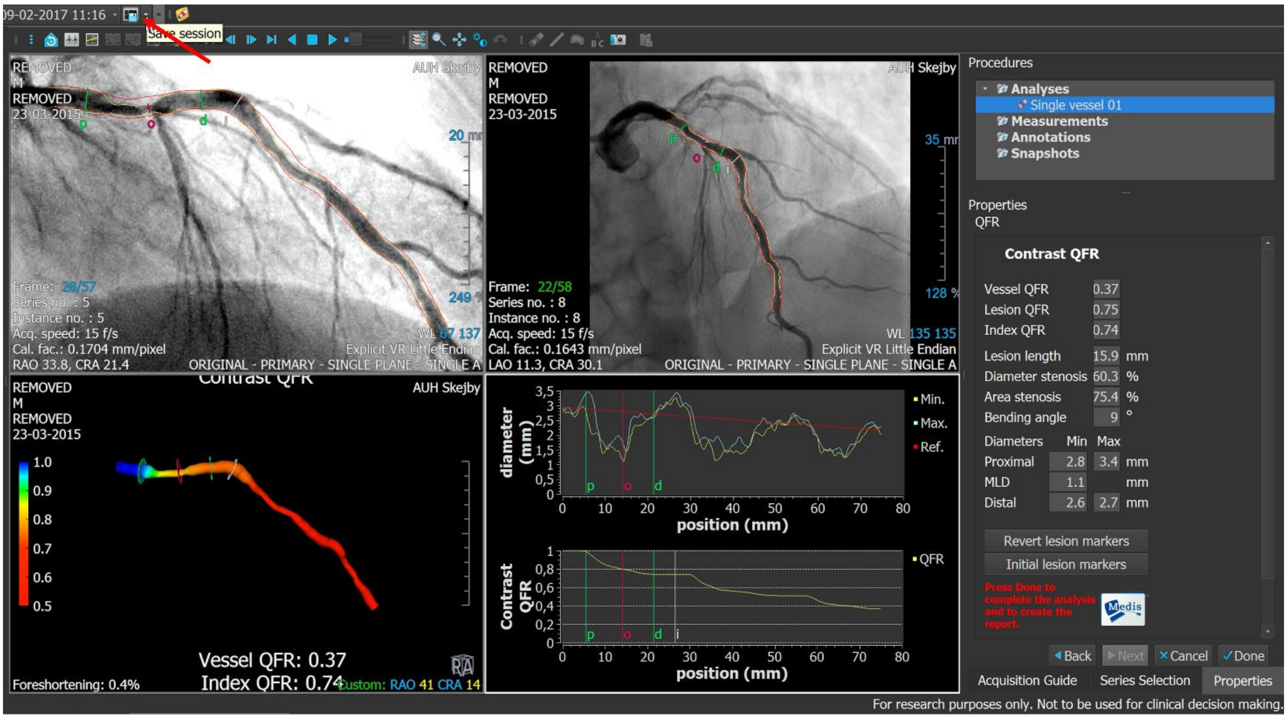

Figure 16 *Documentation. How to save the analysis in the Medis Suite QAngio XA 3D/QFR solution.*

- After an analysis is finalized, Medis Suite QAngio XA 3D/QFR solution creates a report summarizing the analysis, including 2D images of the vessel reconstruction, the 3D reconstruction, results and more (fig. 17).

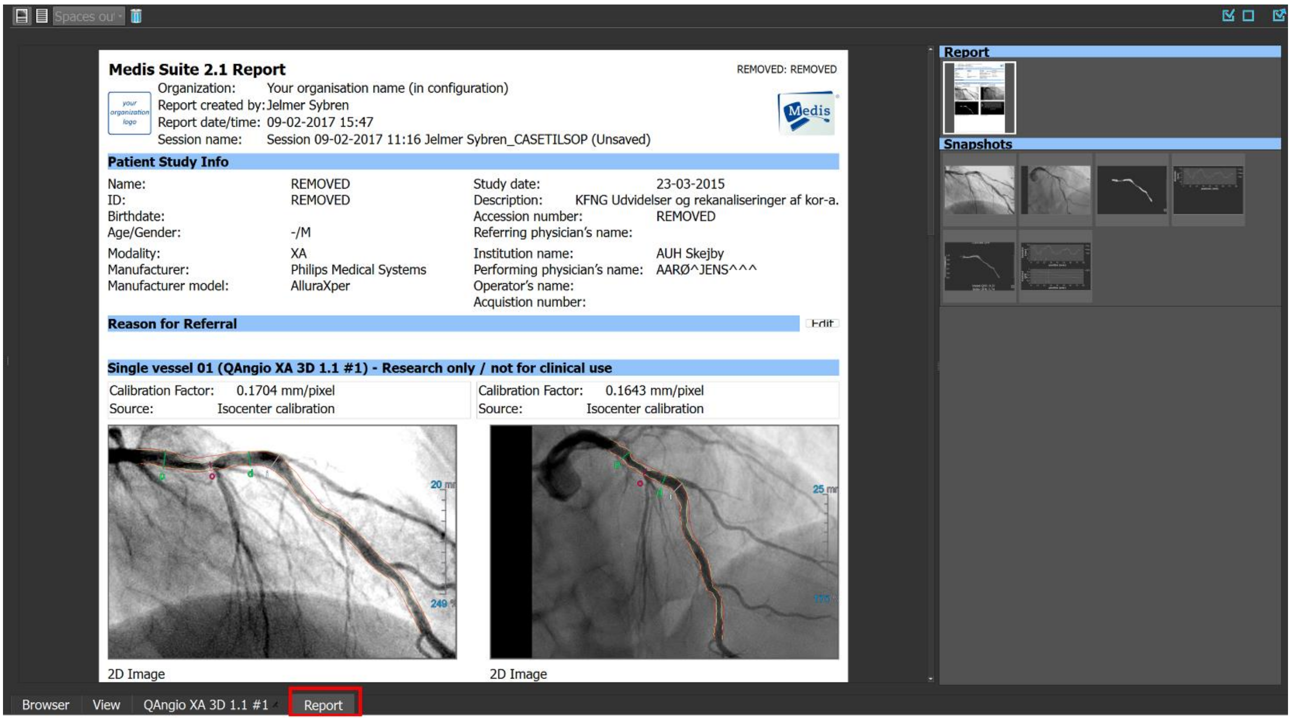

Figure 17 *Report. Access the report by selecting the Report pane (red box).*

3. Specific lesion subsets

Left main coronary artery (LMCA)

- Stenosis in LMCA can only be assessed if the aorto-ostium is not involved (see 1.1)
- QFR of LMCA stenosis in combination with proximal Cx stenosis is not recommended with the present version of QFR (see 1.5)

Ostial stenosis in Cx or SBs with healthy main vessel but large diameter difference

- See 1.5 if the main vessel is diseased, otherwise the following applies:
    - The ostium must be visible in both angiographic views to be able to segment the entire stenosis
    - The proximal marker should be placed in the ostium
    - It is important to optimize the size of the reference diameter. In most cases, reference diameter editing is required (2.8.1 + 2.8.2) to ensure tapering of the reference diameter.

Left main coronary artery (LMCA) + Left anterior descending artery (LAD)

- If a stenosis is present in both the LMCA and LAD, it is important to place the proximal point in the LMCA, proximal to both stenoses

Left anterior descending artery (LAD)

- In QFR analysis of a proximal LAD stenosis in combination with a healthy LMCA, consider to use reference function editing to ensure a fitting to the LAD reference diameter (2.8.1 + 2.8.2

Table S1. Analysis strategy.

		Definition
**Primary comparison**	Sensitivity and specificity of QFR compared to % DS 2D-QCA with FFR as reference	Sensitivity: proportion of true positives. Specificity: proportion of true negatives. QFR and FFR cut-point: ≤ 0.80. Percent DS = ≤ 50 %
**Key secondary analysis**	Feasibility	Fraction of lesions with succesfull FFR measurements where QFR is computed
	Time to QFR	Time from start of frame selection for 3D-reconstruction until QFR based on contrast flow is obtained
	Time to FFR	From introducing the pressure wire in the guiding catheter until verification of drift within limits
	QFR limits to model a QFR-FFR hybrid approach	QFR values to yield a sensitivity and specificity > 90 % and QFR limits to yield a sensitivity and specificity > 95 %

QFR indicates quantitative flow ratio; %DS: percent diameter stenosis; 2D-QCA: two-dimensional quantitative coronary angiography and FFR indicates fractional flow reserve.

Table S2. Inclusion and exclusion criteria.

	Inclusion criteria	Exclusion criteria
**Patient specific**	Stable angina pectoris or secondary evaluation of non-culprit lesion after acute MI	Severe asthma or severe chronic obstructive pulmonary disease
	Age≥18	Acute MI < 72 hours
	Able to provide written informed consent	Severe heart failure (NYHA≥III)
		S-creatinine > 150 umol/L
		Allergy to contrast media or adenosine
		Atrial fibrillation
**Angiographic**	Diameter stenosis 30-90% by visual estimation	Aorta-ostial stenosis
	Reference vessel size >2.0 mm in stenosed segment	Bifurcation stenosis with lesions on both sides of a major shift (>1mm) in reference diameter
		Poor image quality precluding contour detection
		Good contrast filling not possible
		Severe overlap over stenosed segments
		Severe tortuosity of target vessel

MI indicates myocardial Infarction and NYHA indicates York Heart Association Functional Classification.

Table S3. Recommended angulations.

Vessel/Bifurcation	1st View	2nd View
**LM+LAD/LCX**	RAO 20, Caudal 45	AP, Caudal 10
**LAD/Diag**	AP, Cranial 45	RAO 35, Cranial 20
**LCX/OM**	LAO 10, Caudal 25	RAO 25, Caudal 25
**Proximal and mid RCA**	LAO 45, Caudal 0	AP, Caudal 0
**PLA/PDA**	LAO 45, Caudal 0	LAO 30, Caudal 30

Table S4. Number of included patients per site.

Site	Included patients	Patients in analysis
Skejby	116	94
Essen	5	5
Ferrara	48	37
Mestre	5	4
Caserta	16	12
Hague	21	18
Gifu	44	42
Warsaw	36	35
Madrid	12	12
Naples	18	13
Giessen	8	0
**Total**	**329**	**272**

Table S5. Diagnostic accuracy.

A

QFR	FFR		
	<=0.80	>0.80	
<=0.80	90	28	**118**
>0.80	14	185	**199**
	**104**	**213**	**317**

2D-QCA	FFR		
	<=0.80	>0.80	
>=50% DS	46	50	**96**
<50% DS	58	163	**221**
	**104**	**213**	**317**

B

QFR	FFR		
	<=0.80	>0.80	
<=0.80	86	28	**114**
>0.80	17	141	**158**
	**103**	**169**	**272**

2D-QCA	FFR		
	<=0.80	>0.80	
>=50% DS	42	44	**86**
<50% DS	61	125	**186**
	**103**	**169**	**272**

2*2 tables for QFR vs. FFR and QFR vs. 2D-QCA on per-vessel level **(A)** and per-patient level **(B).**

Table S6. Predictors of QFR-FFR discrepancy.

	Multilevel mixed-effect model		Absolute QFR-FFR difference			Correlation	
	Odds Ratio	P	Yes	No	p	Spearman	p
Diabetes	2.88 (95%CI: 1.30-6.43)	0.01	0.03 (IQR: 0.02-0.05)	0.03 (IQR: 0.02-0.07)	0.06		
Smoking	0.44 (95%CI: 0.18-1.03)	0.06	0.03 (IQR: 0.01-0.05)	0.03 (IQR: 0.02-0.06)	0.09		
% DS (2D-QCA)	1.02 (95%CI: 0.98-1.06)	0.23				0.07	0.18

% DS indicates percent diameter stenosis and 2D-QCA indicates two-dimensional diameter stenosis

Figure S1. Vessel-level flowchart.

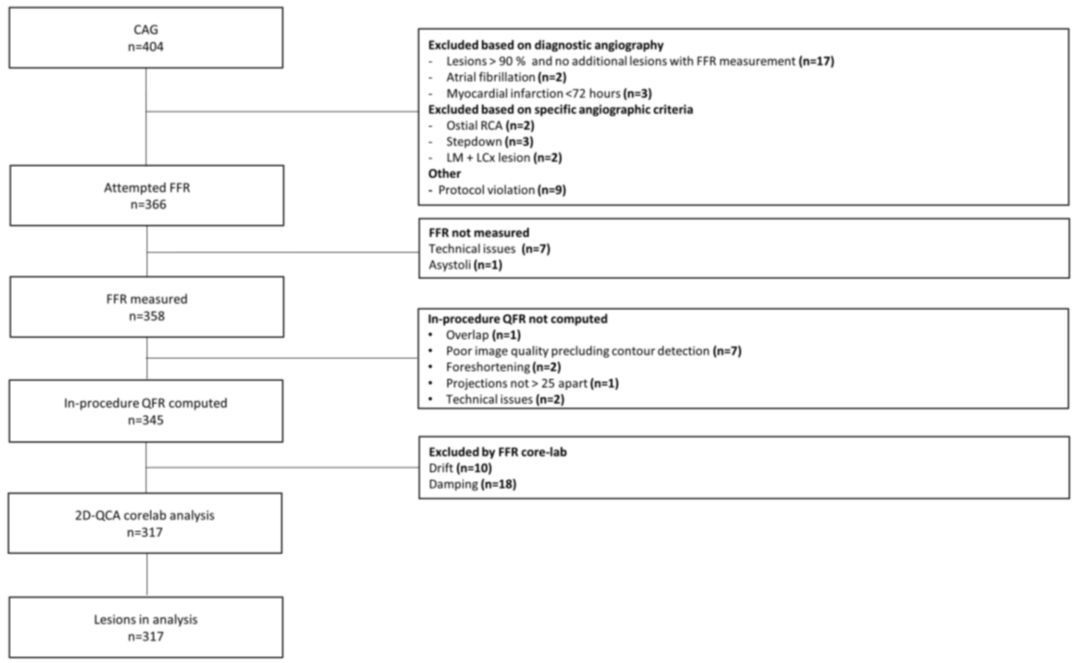

n indicates number of vessel; FFR: fractional flow reserve; RCA: right coronary artery; QFR: quantitative Flow ratio and QCA indicates quantitative coronary angiography.

Figure S2. FFR distribution.

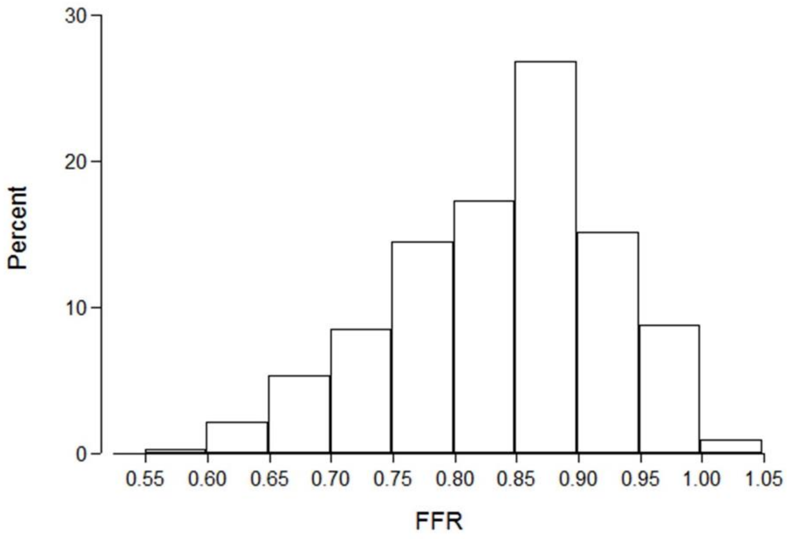

Disease severity according to fractional flow reserve (FFR). Mean FFR was 0.83±0.09 and 101 (32%) lesions were in the 0.75-0.85 interval.

Figure S3. Per-patient level diagnostic performance.

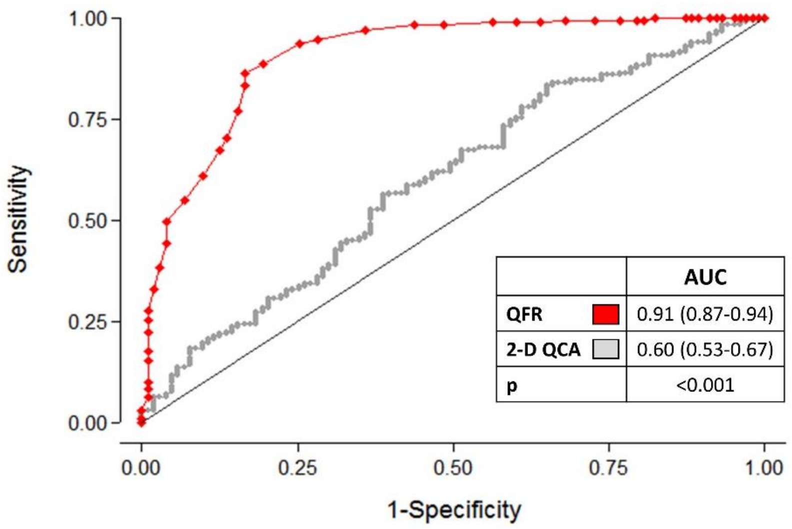

Comparison of quantitative flow ratio (QFR) and two-dimensional quantitative coronary angiography (2D-QCA) using FFR≤0.80 as reference. AUC indicates area under the receiver curve.

Figure S4. Per-patient level correlation and agreement of QFR and FFR.

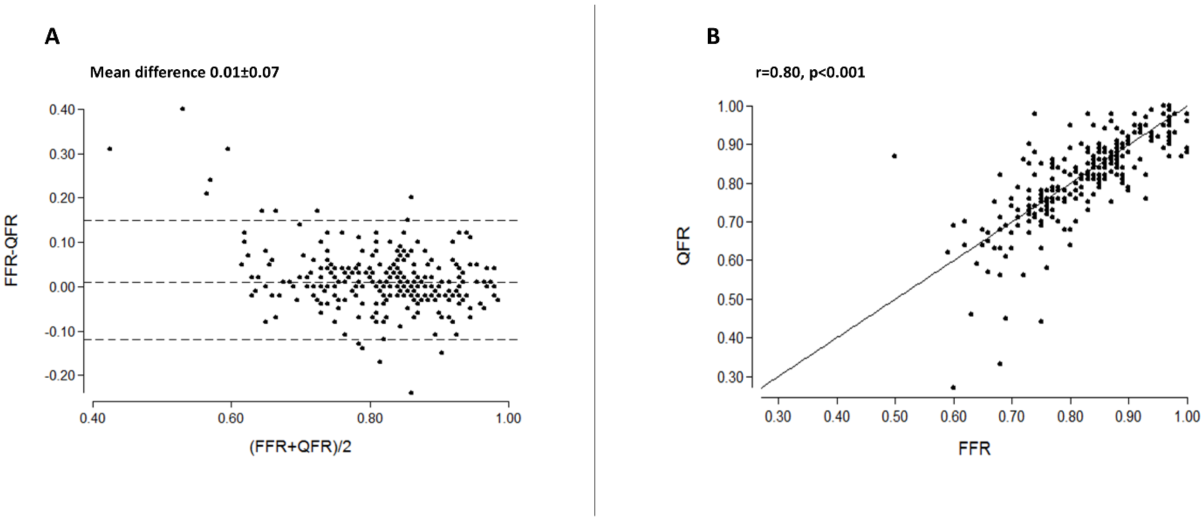

Good per-patient correlation **(A)** and agreement **(B)** of QFR and FFR was observed. Dashed lines in Bland-Altman plot illustrate mean difference ± 2 SD. QFR indicates quantitative flow ratio and FFR indicates fractional flow reserve.

Figure S5. Core-lab QFR correlation and agreement with FFR.

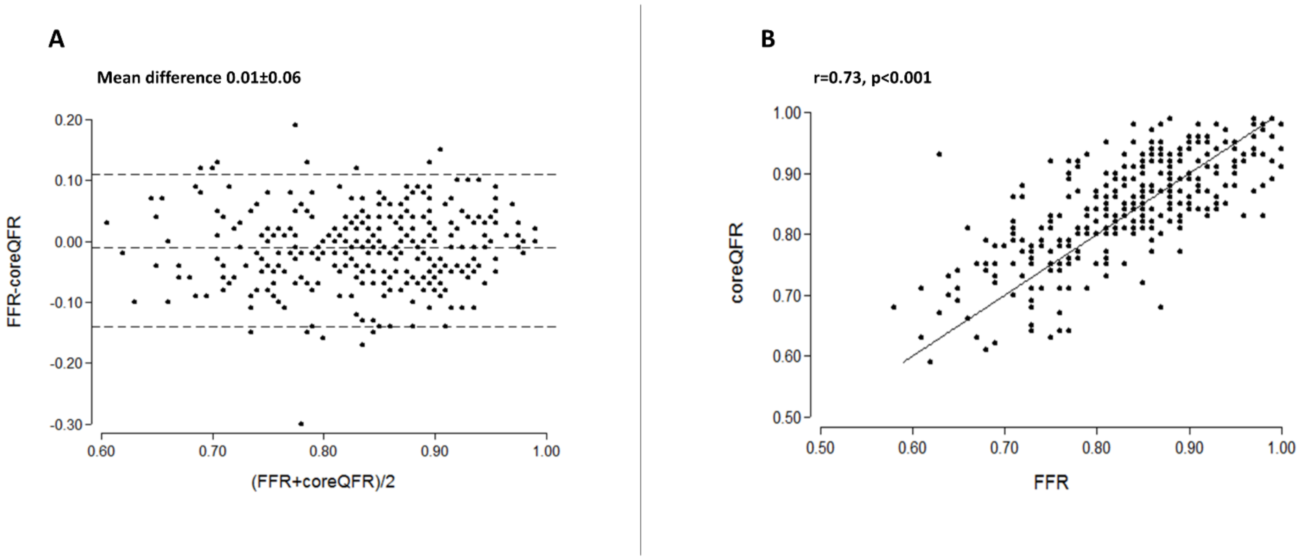

Good correlation **(A)** and agreement **(B)** of between core-lab QFR and FFR was observed. Dashed lines in Bland-Altman plot illustrate mean difference ± 2 SD. QFR indicates quantitative flow ratio and FFR indicates fractional flow reserve.

Figure S6. Per-patient level diagnostic performance of corelab-QFR and in-procedure QFR.

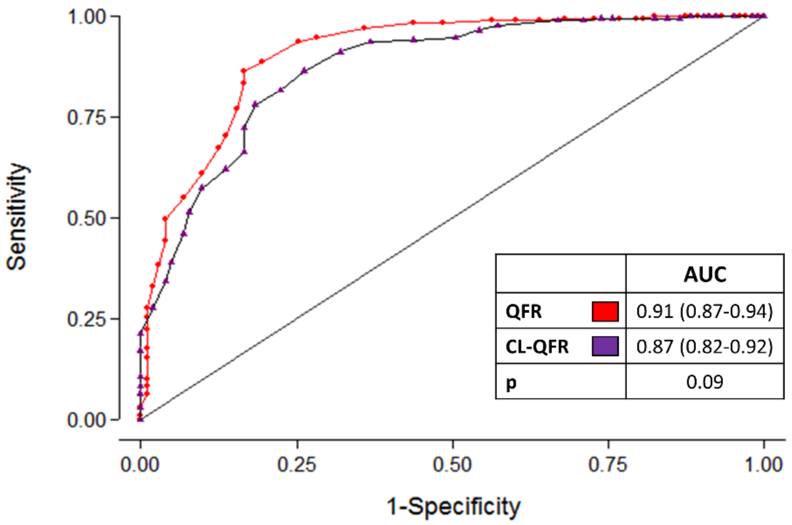

Comparison of in-procedure quantitative flow ratio (QFR) and corelab QFR using FFR≤0.80 as reference. AUC indicates area under the receiver curve.

Figure S7. Correlation and agreement of corelab-QFR and in-procedure QFR.

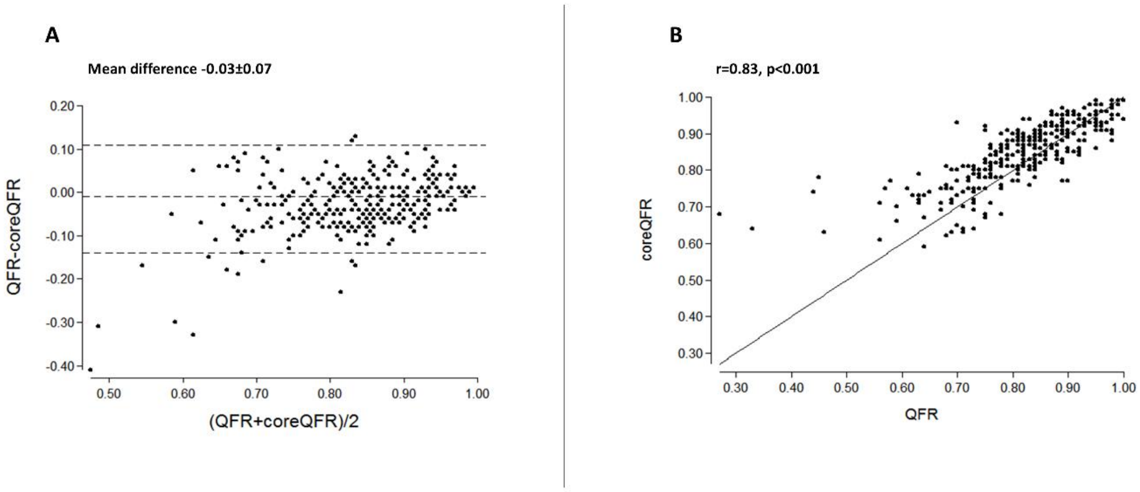

Good correlation **(A)** and agreement **(B)** of in-procedure QFR and corelab QFR was observed. Dashed lines in Bland-Altman plot illustrate mean difference ± 2 SD. QFR indicates quantitative flow ratio and FFR indicates fractional flow reserve.

Figure S8. Clinical application of QFR.

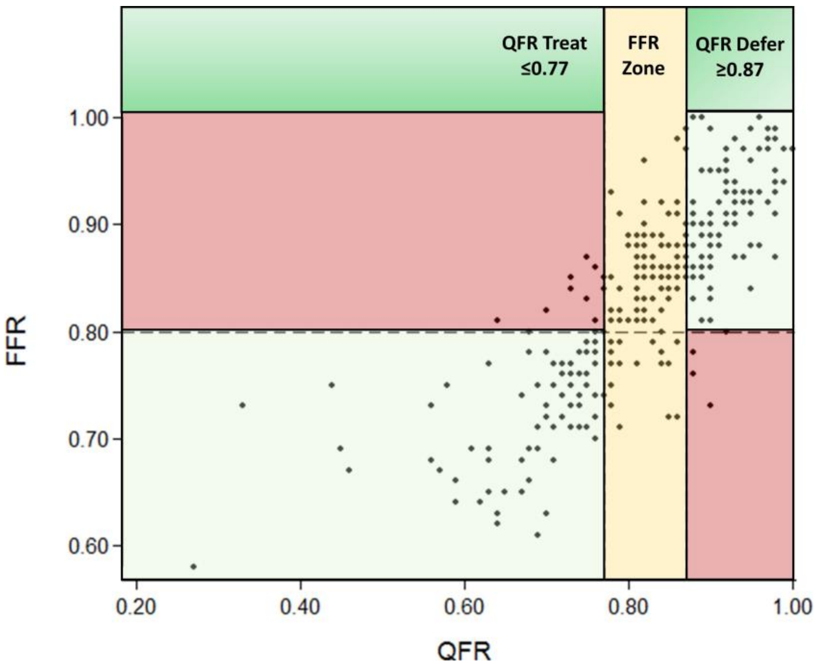

QFR limits to achieve ≥ 95% sensitivity (QFR-treat 0.77) and ≥ 95% specificity (QFR-defer 0.87) were identified for use in a QFR-FFR hybrid approach. QFR indicates quantitative flow ratio and FFR indicates fractional flow reserve.

Figure S9. QFR-FFR hybrid strategy.

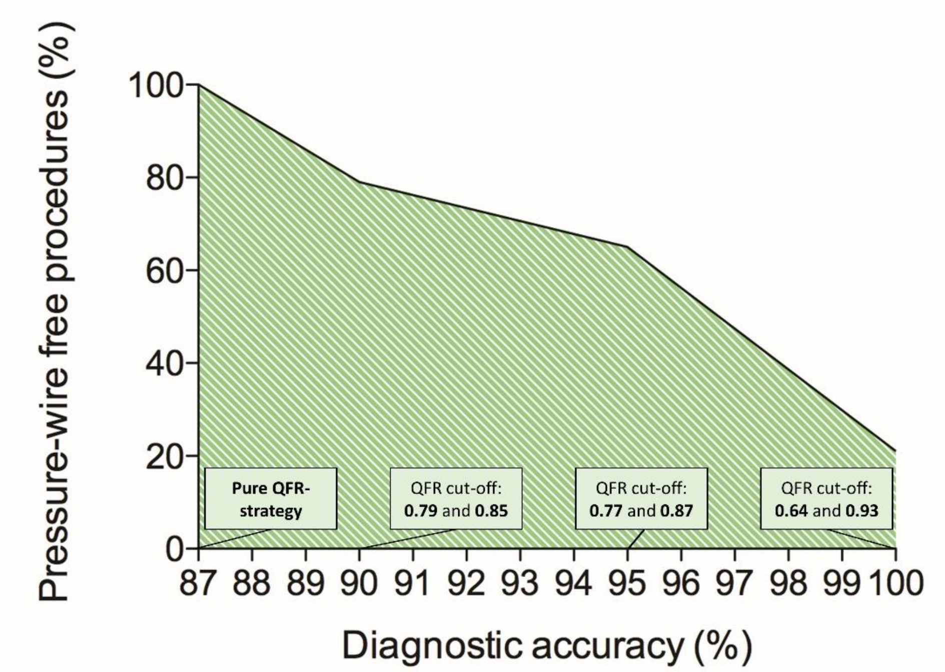

The diagnostic agreement between FFR and QFR increases with adjusted QFR-treat and QFR-defer limits. With increasing diagnostic agreement, fewer lesions are evaluated without pressure-wires and adenosine. This analysis assumes that FFR is 100% accurate. QFR indicates quantitative flow ratio.

Figure S10. Per-vessel level diagnostic performance of in-procedure QFR, 3D-QCA and 2D-QCA.

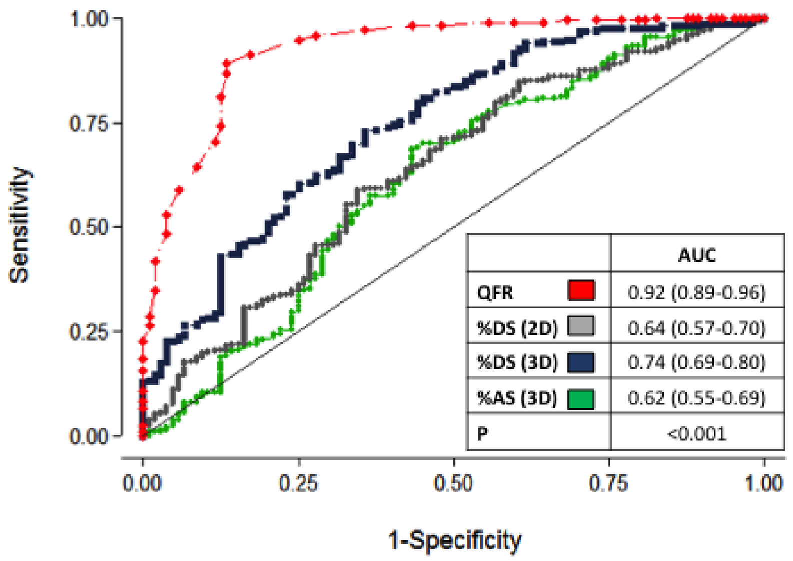

Comparison of in-procedure quantitative flow ratio (QFR), 2D-QCA and 3D-QCA using FFR≤0.80 as reference. AUC indicates area under the receiver curve; %DS: percent diameter stenosis and %AS indicates percent area stenosis.
